# Biomarkers for differentiating diabetic periodontitis from chronic periodontitis: a systematic review and meta-analysis

**DOI:** 10.3389/fimmu.2026.1758079

**Published:** 2026-06-10

**Authors:** Li Zhang, Ru Li, Shengnan Zhang, Wenhui Kong, Peipei Zhang, Wenyue Zhang, Jing Sun

**Affiliations:** 1 Center for Esthetic Dentistry, Jinan Stomatological Hospital, Jinan, Shandong, China; 2 Department of Prosthodontics, Jinan Stomatological Hospital, Jinan, Shandong, China; 3 Jinan Stomatological Hospital, Jinan, Shandong, China; 4 School of Stomatology, Shandong Medical and Pharmaceutical University, Yantai, Shandong, China; 5 Department of Endodontics, Jinan Stomatological Hospital, Jinan, Shandong, China; 6 Hospital Infection Management Office, Jinan Stomatological Hospital., Jinan, Shandong, China; 7 Department of Oral Surgery, Jinan Stomatological Hospital Shungeng Branch, Jinan, Shandong, China; 8 Central Laboratory, Jinan Key Medical and Health Laboratory of Oral Diseases and Tissue Regeneration, Jinan Key Laboratory of Oral Diseases and Tissue Regeneration, Shandong Provincial Key Medical and Health Laboratory of Oral Diseases and Tissue Regeneration, Jinan Stomatological Hospital, Jinan, Shandong, China

**Keywords:** biomarkers, chronic periodontitis, diabetes-related periodontitis, meta-analysis, systematic review

## Abstract

**Background:**

Diabetes-related periodontitis (DMCP) and chronic periodontitis (CP) are associated with significant differences in clinical presentation, pathogenesis, and treatment outcomes. However, reliable biomarkers for their early differentiation remain elusive. This study aims to identify discriminatory biomarkers between DMCP and CP to provide an evidence-based foundation for improved clinical management.

**Methods:**

A systematic search of PubMed, Embase, the Cochrane Library, and Web of Science was conducted from their inception until October 2025 for studies comparing biomarkers in DMCP and CP. Differences in biomarker levels between the two groups were expressed as Standardized Mean Differences (SMD) with 95% Confidence Intervals (CI). All meta-analyses were performed using a random-effects model.

**Results:**

A total of 33 eligible studies were included, involving 970 patients with DMCP and 905 with CP. The meta-analysis revealed the following: (1) lipid profiles: the DMCP group demonstrated significantly higher levels of very low-density lipoprotein (VLDL) (SMD: 0.91; 95%CI: 0.43 to 1.39; *P*<0.001) compared to the CP group; (2) inflammatory factors: levels of interleukin-8 (IL-8) (SMD: 0.38, 95%CI: 0.06 to 0.70; P=0.021), and high-sensitivity C-reactive protein (hs-CRP) (SMD: 2.56; 95%CI: 0.31 to 4.82; P=0.026) were significantly elevated in the DMCP group; (3) oxidative stress-related markers: A significant decrease was observed in catalase (CAT) (SMD: -0.31; 95%CI: -0.58 to -0.05; P=0.021) and oxidized glutathione (GSSG) (SMD: -1.16; 95%CI: -1.76 to -0.55; P < 0.001) in the DMCP group. Conversely, levels of nitric oxide synthase (NOS) (SMD: 0.58; 95%CI: 0.11 to 1.05; P=.016) and 4-hydroxynonenal (4-HNE) (SMD: 7.05; 95%CI: 5.07 to 9.03; P < 0.001) were markedly higher; and (4) other biomarkers: the DMCP group exhibited significantly elevated levels of body mass index, eotaxin, glucose-dependent insulinotropic polypeptide, glucagon-like peptide-1, and plasminogen activator inhibitor-1, alongside significantly reduced levels of C-peptide and 25-hydroxyvitamin D.

**Conclusions:**

Significant differences in biomarkers related to lipid metabolism, inflammatory response, and oxidative stress were observed between patients with DMCP and CP. Key indicators demonstrating relatively robust differences include VLDL, 4-HNE, CAT, GSSG, NOS, IL-8, hs-CRP, and C-peptide.

**Systematic Review Registration:**

https://inplasy.com/inplasy-2025-11-0067/, identifier INPLASY2025110067.

## Introduction

Periodontitis is a chronic inflammatory disease triggered by dental plaque biofilm. Its core pathological process involves the progressive destruction of periodontal supporting tissues, which can lead to tooth loosening and loss in advanced stages, making it a leading cause of adult tooth loss worldwide (1). As the understanding of the disease spectrum has evolved, increasing attention has been drawn to the relationship between periodontitis and systemic conditions. Among these, the well-established bidirectional relationship between diabetes and periodontitis has become a key area of research (2). Poor glycemic control exacerbates periodontitis through multiple mechanisms: hyperglycemia promotes the formation of advanced glycation end products (AGE), which trigger pro-inflammatory cytokine release via the receptor for advanced glycation end products (RAGE) pathway; diabetes-induced oxidative stress impairs neutrophil function and delays tissue repair; and microvascular dysfunction compromises periodontal tissue perfusion and immune surveillance (3, 4). Conversely, periodontal inflammation contributes to systemic insulin resistance through the release of inflammatory mediators such as interleukin-6 (IL-6) and tumor necrosis factor-alpha (TNF-α) into the circulation, which interfere with insulin signaling pathways, thereby creating a vicious cycle (5, 6). According to the 2017 World Workshop on the Classification of Periodontal and Peri-Implant Diseases and Conditions, periodontitis is classified into three categories: periodontitis, necrotizing periodontitis, and periodontitis as a manifestation of systemic conditions (7). Within this framework, diabetes is recognized as an important modifying factor rather than as a distinct diagnostic entity. In this review, we use the term “diabetes-related periodontitis (DMCP)” descriptively to refer to periodontitis occurring in patients with diabetes, acknowledging that this is not a separate diagnostic category but rather periodontitis modified by the diabetic state. Patients with periodontitis and diabetes typically present with more pronounced inflammation, accelerated progression, and less favorable treatment responses compared to periodontitis patients without diabetes.

In its early stages, DMCP lacks clearly distinguishable clinical features from periodontitis in patients without diabetes (referred to as CP, acknowledging that under the 2017 classification both groups fall under the single category of “periodontitis”). Both conditions commonly manifest as gingival redness, bleeding, periodontal pocket formation, and alveolar bone resorption, complicating early clinical differentiation based on presentation alone (8). However, the presence of diabetes as a modifying factor may influence disease progression and treatment outcomes. This diagnostic uncertainty has implications for treatment planning. Patients with DMCP require integrated management combining glycemic control and anti-inflammatory periodontal therapy, whereas CP primarily focuses on eliminating local infectious sources. Misdiagnosis may lead to inappropriate treatment regimens, which could reduce therapeutic efficacy and delay systemic disease management (9). Therefore, identifying specific biomarkers for early and accurate differentiation between these two forms of periodontitis holds significant clinical relevance for optimizing treatment strategies and improving patient prognosis.

Biomarkers, as measurable indicators of pathological processes or disease states, are widely utilized in diagnosis, differentiation, and prognostic assessment owing to their specificity and detectability (10, 11). Nevertheless, in the context of differentiating periodontitis subtypes, a comprehensive synthesis and quantitative evaluation of proposed biomarkers is lacking. This study aims to synthesize available evidence on biomarkers that differentiate DMCP from CP, with the goal of identifying those with robust discriminatory power for clinical application.

## Materials and methods

### Data sources, search strategy, and selection criteria

This systematic review and meta-analysis was conducted in accordance with the Preferred Reporting Items for Systematic Reviews and Meta-Analyses (PRISMA) guidelines (12). The study protocol was prospectively registered on the INPLASY platform (Registration number: INPLASY2025110067), specifying the research objectives, eligibility criteria, data extraction plan, and statistical analysis methods *a priori* to minimize selection bias. A comprehensive literature search was performed from inception to October 2025 across four databases: PubMed, Embase, the Cochrane Library, and Web of Science. The search strategy combined Medical Subject Headings (MeSH) with free-text terms, using core keywords such as “diabetic periodontitis,” “diabetes mellitus periodontitis,” and “chronic periodontitis.” The detailed search strategy for electronic databases is provided in **Supplementary File 1**. Additionally, the reference lists of included studies were manually screened. We also searched conference proceedings, dissertations, and unpublished studies to identify other potentially eligible records. When data were incomplete or required clarification, the original authors were contacted via email to obtain supplementary information.

Literature screening was independently performed by two investigators based on predefined criteria. The process involved reviewing titles and abstracts to exclude obviously irrelevant records. The full texts of potentially eligible studies were then retrieved and thoroughly reviewed for final inclusion. Any disagreements between the two reviewers were resolved through discussion or, if necessary, by consultation with a third senior researcher. The inclusion criteria were as follows: (1) Population: patients with a confirmed diagnosis of DMCP and patients with CP; (2) Exposure/Outcome: studies that measured and reported the expression levels of biomarkers in both patient groups; (3) Study type: published cross-sectional, case-control, or cohort studies; (4) Data availability: studies that provided complete data (e.g., sample size, mean, standard deviation, median, interquartile range) or from which these data could be extracted or calculated; and (5) Language: studies published in Chinese or English.

### Data collection and quality assessment

A standardized data extraction form was developed. Two investigators independently extracted key information from the included studies, including the first author, publication year, study design, country/region, sample size in each group, age, sex distribution, smoking status, hemoglobin A1c (HbA1c), duration of diabetes, or treatment status, biomarker names, and outcome data. The methodological quality of the included studies was assessed using the Newcastle-Ottawa Scale (13). The Newcastle-Ottawa Scale evaluates studies across three domains: selection of study groups (maximum 4 stars), comparability of groups (maximum 2 stars), and ascertainment of exposure/outcome (maximum 3 stars). A star rating of 7–9 indicated high quality, 5–6 moderate quality, and ≤4 low quality. Two reviewers independently appraised the quality of each study. Their results were cross-checked, and any disagreements were resolved through discussion or by consulting a third reviewer.

### Statistical analysis

Differences in biomarker levels between the two patient groups were expressed as the Standardized Mean Difference (SMD) with a 95% Confidence Interval (CI). A random-effects model was employed for all meta-analyses to account for potential heterogeneity among the included studies (14, 15). Heterogeneity was assessed using the Cochran’s Q-test and the I² statistic, with significant heterogeneity defined as a P-value < 0.10 for the Q-test or an I² statistic ≥ 50% (16, 17). Sensitivity analysis was performed by sequentially excluding each individual study and recalculating the pooled effect size to evaluate the robustness of the results (18). Publication bias was evaluated using funnel plots, complemented by Egger’s or Begg’s test. Symmetrical funnel plots and a test P-value ≥ 0.05 indicated a low risk of publication bias. If significant publication bias was detected, the trim-and-fill method was applied for adjustment (19–21). A two-sided alpha level of 0.05 was set for all statistical tests. All analyses were performed using STATA 15.0 (StataCorp, College Station, TX, USA).

## Results

### Literature search

**Figure 1:**
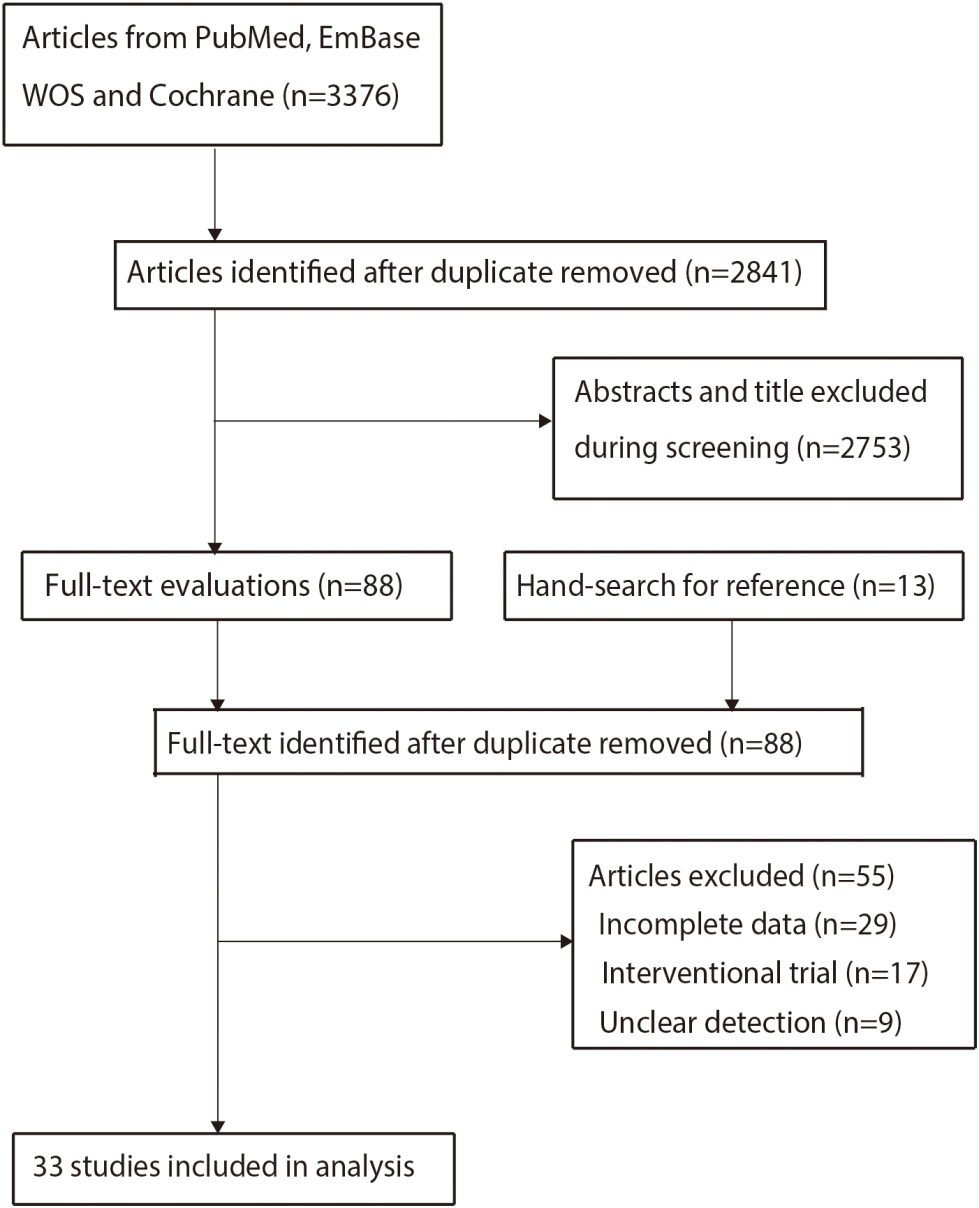
Flow diagram of study selection process.

The initial search identified 3,376 potentially relevant records. After removing duplicates, 2,841 records underwent initial screening based on titles and abstracts, leading to the exclusion of 2,753 records that did not meet the inclusion criteria. The full texts of the remaining 88 citations were retrieved and thoroughly assessed. Of these, 55 were excluded for the following reasons: incomplete data (n=29), interventional trial design (n=17), and unclear detection methods (n=9). Consequently, 33 studies were included in the final analysis (22–54). The study selection process is detailed in **Figure 1**.

### Study characteristics

The 33 included studies, all cross-sectional in design, were published between 2008 and 2024. They were primarily conducted in Asia, with others from Europe, the Americas, and Africa. These studies involved a total of 1,875 participants, comprising 970 patients with DMCP and 905 patients with CP, with individual study sample sizes ranging from 16 to 200. All studies focused on type 2 diabetes. The basic characteristics of the included studies are summarized in **Table 1**. Only 4 of 33 studies (12.1%) reported baseline smoking status data. We attempted to extract data on HbA1c, diabetes duration, and antidiabetic treatment status from all 33 included studies to assess potential confounding by glycemic status. However, the majority of studies did not report these parameters in a format suitable for subgroup analysis or statistical adjustment. Specifically, only 11 studies reported mean HbA1c values for the DMCP group, and none provided individual-level HbA1c data or stratified biomarker analyses by glycemic control categories. Six studies reported diabetes duration, and two studies described treatment status (oral hypoglycemic agents vs. insulin). Quality assessment using the Newcastle-Ottawa Scale indicated that all included studies were of moderate quality, with scores ranging from 5 to 6.

**Table 1:** The baseline characteristics of included studies and involved patients.

Study	Study design	Country	Sample size (DMCP/CP)	Age (years)	Male (%)	Smoking status (%)	HbA1c (%)	Duration of DM (years)	Treatment status	Reported outcomes	NOS
Akalın 2008	Cross-sectional	Turkey	17/17	51.8/49.4	55.9	NA	NA	NA	NA	SOD, HDL, LDL, VLDL, TC, TG	5
Gumus 2009	Cross-sectional	Turkey	25/24	54.4/37.1	42.9	28.6	7.2	NA	NA	GSH, GSSG, TAOC, UA	5
Kardesler 2010	Cross-sectional	Turkey	25/15	52.9/51.3	67.5	35.0	NA	7.1	NA	TC, TG, LDL, HDL, BMI	5
Allen 2011	Cross-sectional	Ireland	20/20	56.0/54.0	70.0	NA	NA	7.0	Oral hypoglycaemics (95%)	SMAC, leucocytes, neutrophils, lymphocytes, fibrinogen, hsCRP, TC, TG, VLDL, HDL, LDL	6
Ribeiro 2011	Cross-sectional	Brazil	37/20	52.5/51.5	42.1	NA	9.0	6.4	NA	IL-4, IFN-γ, IL-17, IL-23, TNF-α, RANKL, OPG	6
Duarte 2012	Cross-sectional	Brazil	30/15	47.7/49.0	48.9	NA	9.3	6.1	NA	IL-4, IFN-γ, IL-10, IL-17, IL-23, IL-6, TNF-α, TGF-β, Foxp3, RORC2, RANKL, OPG, TLR-2, TLR-4, RAGE	6
Pradeep 2012	Cross-sectional	India	10/10	38.8/32.6	50.0	NA	NA	NA	NA	Visfatin, BMI	5
Pendyala 2013	Cross-sectional	India	30/30	40.0-65.0	41.7	NA	NA	NA	NA	BMI, TC, TG, HDL, LDL, TAOC	5
Thomas 2013	Cross-sectional	India	50/50	NA	NA	NA	NA	NA	NA	GSH, CAT	6
Jung 2013	Cross-sectional	Korea	16/16	NA	NA	NA	NA	NA	NA	NOS, TIMP-3, TIMP-4	5
Shaker 2013	Cross-sectional	Egypt	20/20	50.1/45.2	45.0	NA	7.7	NA	NA	NOS	6
Pradeep 2013	Cross-sectional	India	15/15	36.6/35.8	NA	NA	NA	NA	NA	4-HNE	5
Kalra 2013	Cross-sectional	India	15/15	32.2/31.5	50.0	NA	NA	NA	NA	SCF, hsCRP	5
Thomas 2014	Cross-sectional	India	50/50	35.0-65.0	NA	NA	NA	NA	NA	TAOC, SOD	6
Trivedi 2014	Cross-sectional	India	30/30	NA	35.0	NA	6.8	5.5	NA	MDA, SOD, CAT, GR	5
Duarte 2014	Cross-sectional	Brazil	26/20	50.3/51.4	71.7	NA	9.6	NA	NA	Eotaxin, IL-8, MCP-1, MIP-1α, GM-CSF, G-CSF, IL-1β, IL-6, TNF-α, IFN-γ,IL-10, IL-12, IL-2, IL-7	5
Sudhakar 2015	Cross-sectional	India	20/20	NA	NA	NA	NA	NA	NA	ROM	5
Mohamed 2015	Cross-sectional	Norway	54/30	54.8/55.4	48.8	17.9	9.2	8.4	Oral hypoglycaemics (66.7%), insulin (29.6%), both (3.7%)	C-peptide, GIP, GLP-1, glucagon, ghrelin, leptin, PAI-1, visfatin	6
Acharya 2015	Cross-sectional	India	15/15	30.0-55.0	NA	NA	NA	NA	NA	IL-10	5
Joseph 2015	Cross-sectional	India	43/50	43.4/40.8	36.6	7.5	NA	NA	NA	BMI, 25(OH)D	6
Ghallab 2015	Cross-sectional	Egypt	20/20	47.5/48.3	50.0	NA	7.2	NA	NA	Leptin, visfatin	5
Patil 2016	Cross-sectional	India	25/25	NA	NA	NA	NA	NA	NA	MDA, TAOC, SOD, CAT	6
Vincent 2018	Cross-sectional	India	20/20	48.9/38.1	52.5	NA	NA	NA	NA	TAOC, TOS, OSI	5
Koregol 2018	Cross-sectional	India	30/30	50.4/45.9	41.7	NA	NA	NA	NA	8-isoprostane	5
Guo 2018	Cross-sectional	China	22/22	62.8/58.1	75.0	NA	NA	NA	NA	BMI, TG, TC, HDL, LDL, ALT, AST, ferritin, hepcidin	6
Acharya 2018	Cross-sectional	India	20/20	44.1/42.1	55.0	NA	NA	NA	NA	TNF-α, IL-4, IL-6, IL-10	5
Bakshi 2018	Cross-sectional	India	15/15	51.0	53.3	NA	7.9	NA	NA	TNF-α, IL-4, IL-6	5
Miranda 2019	Cross-sectional	Brazil	52/53	55.1/52.2	35.2	NA	7.8	NA	NA	IL-10, IL-4, IL-5, IL-13, IL-2, TGF-β, IL-8, MIP-1α, IL-1β, TNF-α, IL-6, IFN-γ, IL-12, IL-17, IL-21, IL-23, GM-CSF, IL-7	6
Ahuja 2019	Cross-sectional	India	30/30	NA	NA	NA	NA	NA	NA	BMI, leptin	5
Shee 2020	Cross-sectional	India	8/8	58.4/42.0	NA	NA	NA	NA	NA	MDA	5
Thomas 2021	Cross-sectional	India	100/100	30.0-60.0	NA	NA	NA	NA	NA	SOD, TAOC, CAT, GSH	6
Sangappa 2024	Cross-sectional	India	60/60	48.9	48.3	NA	NA	NA	NA	IL-6	6
Lobão 2024	Cross-sectional	Brazil	20/20	60.8/51.7	25.0	NA	8.3	NA	NA	BMI, TAOC, uric acid	5

4-HNE, 4-hydroxynonenal; 8-isoprostane, 8-isoprostaglandin F2α; AGE, advanced glycation end product; ALT, alanine aminotransferase; AST, aspartate aminotransferase; BMI, body mass index; CAT, catalase; CI, confidence interval; CP, chronic periodontitis; DM, diabetes mellitus; DMCP, diabetes-related periodontitis; Foxp3, forkhead box P3; G-CSF, granulocyte colony-stimulating factor; GIP, glucose-dependent insulinotropic polypeptide; GLP-1, glucagon-like peptide-1; GM-CSF, granulocyte-macrophage colony-stimulating factor; GR, glutathione reductase; GSH, reduced glutathione; GSSG, oxidized glutathione; HbA1c, hemoglobin A1c; HDL, high-density lipoprotein; hs-CRP, high-sensitivity C-reactive protein; IFN-γ, interferon-gamma; IL, interleukin; LDL, low-density lipoprotein; MCP-1, monocyte chemoattractant protein-1; MDA, malondialdehyde; MIP-1α, macrophage inflammatory protein-1 alpha; NA, not available; NOS, nitric oxide synthase; OPG, osteoprotegerin; OSI, oxidative stress index; PAI-1, plasminogen activator inhibitor-1; RAGE, receptor for advanced glycation end products; RANKL, receptor activator of nuclear factor-kappa B ligand; ROM, reactive oxygen metabolites; RORC2, RAR-related orphan receptor C2; SCF, stem cell factor; SMAC, second mitochondria-derived activator of caspase; SMD, standardized mean difference; SOD, superoxide dismutase; TAOC, total antioxidant capacity; TC, total cholesterol; TG, triglycerides; TGF-β, transforming growth factor-beta; TIMP, tissue inhibitor of metalloproteinase; TLR, toll-like receptor; TNF-α, tumor necrosis factor-alpha; TOS, total oxidant status; UA, uric acid; VLDL, very low-density lipoprotein; 25(OH)D, 25-hydroxyvitamin D.

### Lipid profiles

**Figure 2:**
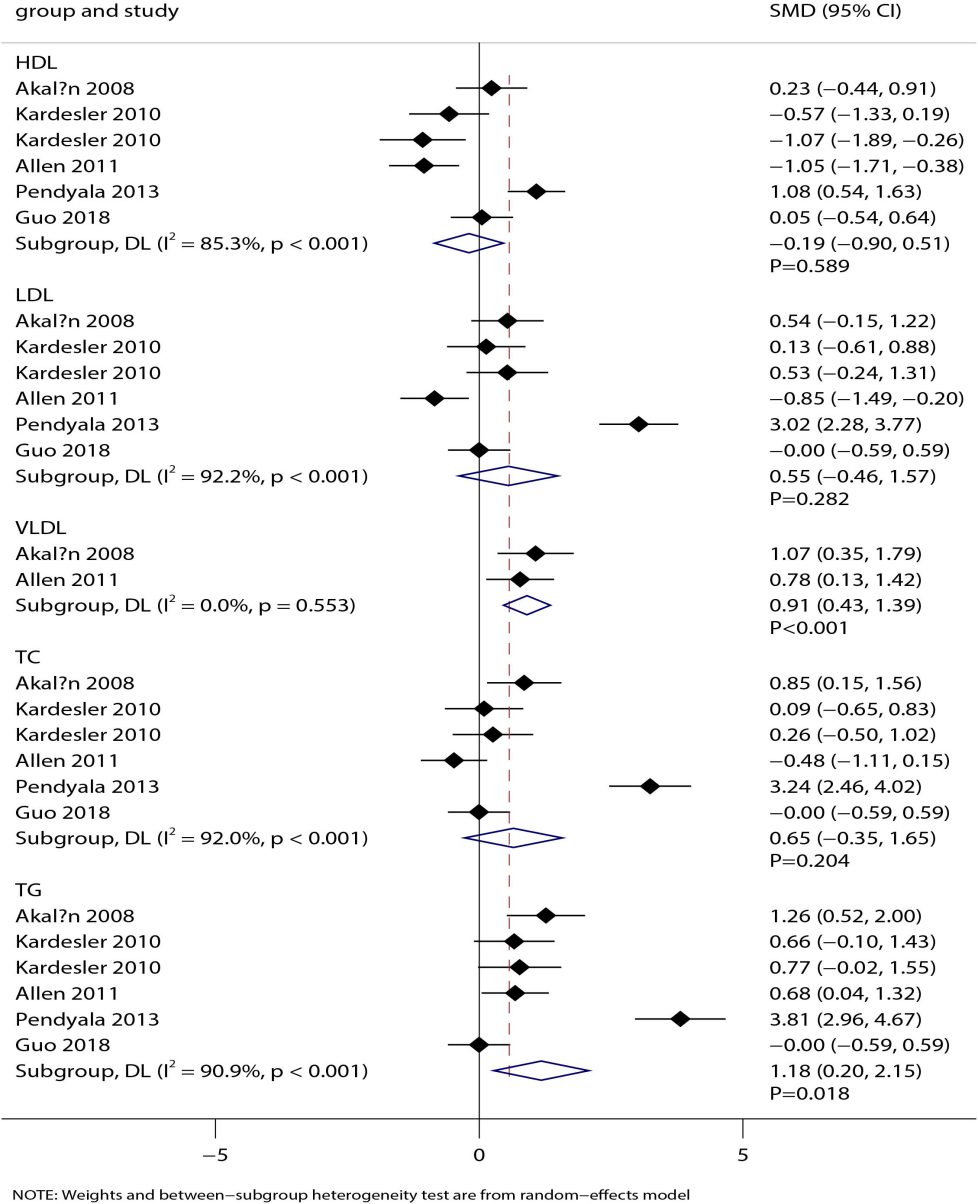
Forest plots of differences in lipid profiles between patients with diabetic-related periodontitis (DMCP) and chronic periodontitis (CP).

**Figure 2** illustrates the differences in lipid profiles between the two groups. Compared with the CP group, DMCP patients were associated with higher levels of very low-density lipoprotein (VLDL) (SMD: 0.91; 95% CI: 0.43 to 1.39; P < 0.001) and triglycerides (TG) (SMD: 1.18; 95% CI: 0.20 to 2.15; P=0.018). In contrast, no significant differences were observed in high-density lipoprotein (HDL), low-density lipoprotein (LDL), or total cholesterol (TC). Heterogeneity analysis revealed no significant heterogeneity for VLDL (I² = 0.0%; P=0.553), whereas significant heterogeneity was detected for HDL (I² = 85.3%; P < 0.001), LDL (I² = 92.2%; P < 0.001), TC (I² = 92.0%; P < 0.001), and TG (I² = 90.9%; P < 0.001). Sensitivity analysis indicated that the non-significant differences for HDL, LDL, and TC were relatively stable, while the significant difference for TG was unstable (**Supplementary File 2**). Publication bias analysis showed no significant publication bias for HDL (Egger’s P=0.058; Begg’s P=0.260), LDL (Egger’s P=0.284; Begg’s P=0.260), TC (Egger’s P=0.193; Begg’s P=0.133), or TG (Egger’s P=0.073; Begg’s P=0.060) (**Supplementary File 3**).

### Inflammatory factors

**Figure 3:**
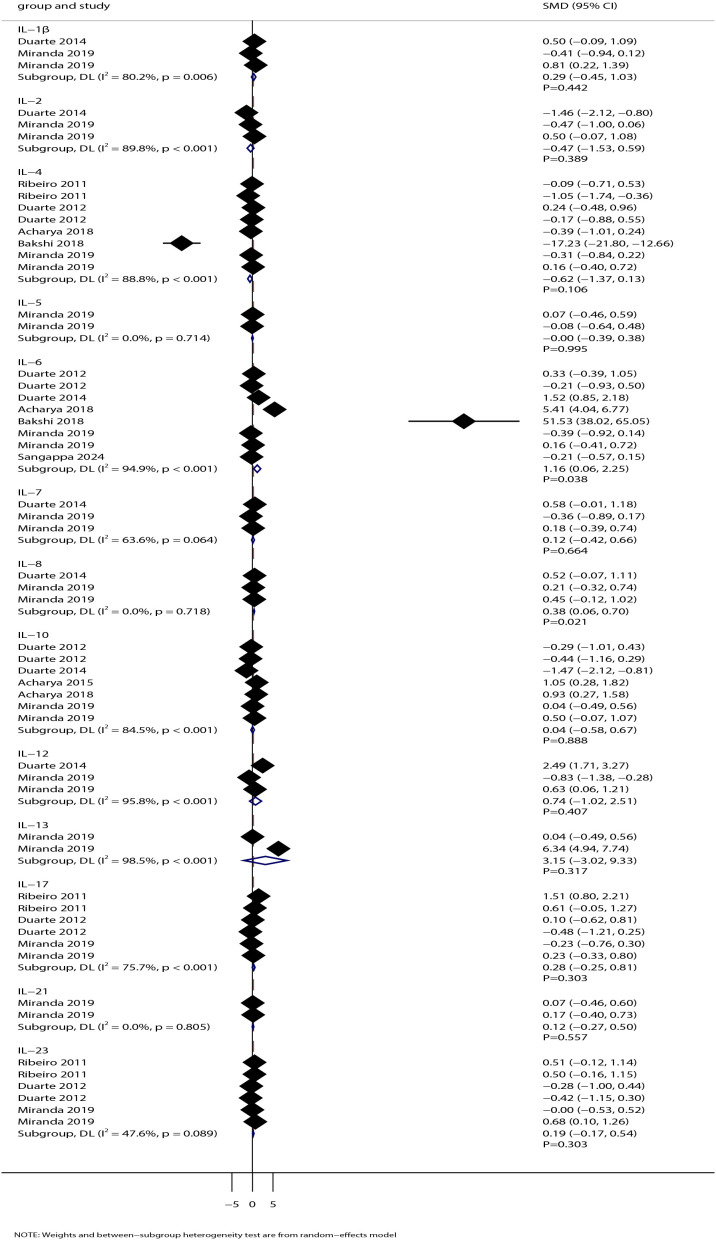
Forest plots of differences in inflammatory factor levels between the DMCP and CP groups.

**Figure 3** illustrates the differences in inflammatory factor levels between the DMCP and CP groups. The analysis revealed that the DMCP group were associated with higher levels of IL-6 (SMD: 1.16; 95% CI: 0.06 to 2.25; P=0.038) and interleukin-8 (IL-8) (SMD: 0.38; 95% CI: 0.06 to 0.70; P=0.021) compared to the CP group. In contrast, no significant intergroup differences were observed for interleukin-1 beta (IL-1β), interleukin-2 (IL-2), interleukin-4 (IL-4), interleukin-7 (IL-7), interleukin-10 (IL-10), interleukin-12 (IL-12), interleukin-13 (IL-13), interleukin-17 (IL-17), interleukin-21 (IL-21), or interleukin-23 (IL-23) (all P > 0.05). Heterogeneity was non-significant for IL-5 (I^2^ = 0.0%; P=0.714), IL-8 (I^2^ = 0.0%; P=0.718), and IL-21 (I^2^ = 0.0%; P=0.805), but significant for IL-1β (I^2^ = 80.2%; P=0.006), IL-2 (I^2^ = 89.8%; P < 0.001), IL-4 (I^2^ = 88.8%; P < 0.001), IL-6 (I^2^ = 94.9%; P < 0.001), IL-10 (I^2^ = 84.5%; P < 0.001), IL-12 (I^2^ = 95.8%; P < 0.001), IL-13 (I^2^ = 98.5%; P < 0.001), IL-17 (I^2^ = 75.7%; P < 0.001), and IL-23 (I^2^ = 47.6%; P=0.089). Sensitivity analysis indicated that the finding for IL-6 was unstable, whereas the results for IL-4 and IL-10 were robust (**Supplementary File 2**). Significant publication bias was detected for IL-4 (Egger’s P=0.003) and IL-6 (Egger’s P=0.005; Begg’s P=0.019), but not for IL-10. After applying the trim-and-fill method, the conclusion for IL-4 remained unchanged. However, the adjusted analysis showed no significant difference in IL-6 levels between the groups (**Supplementary File 3**).

**Figure 4:**
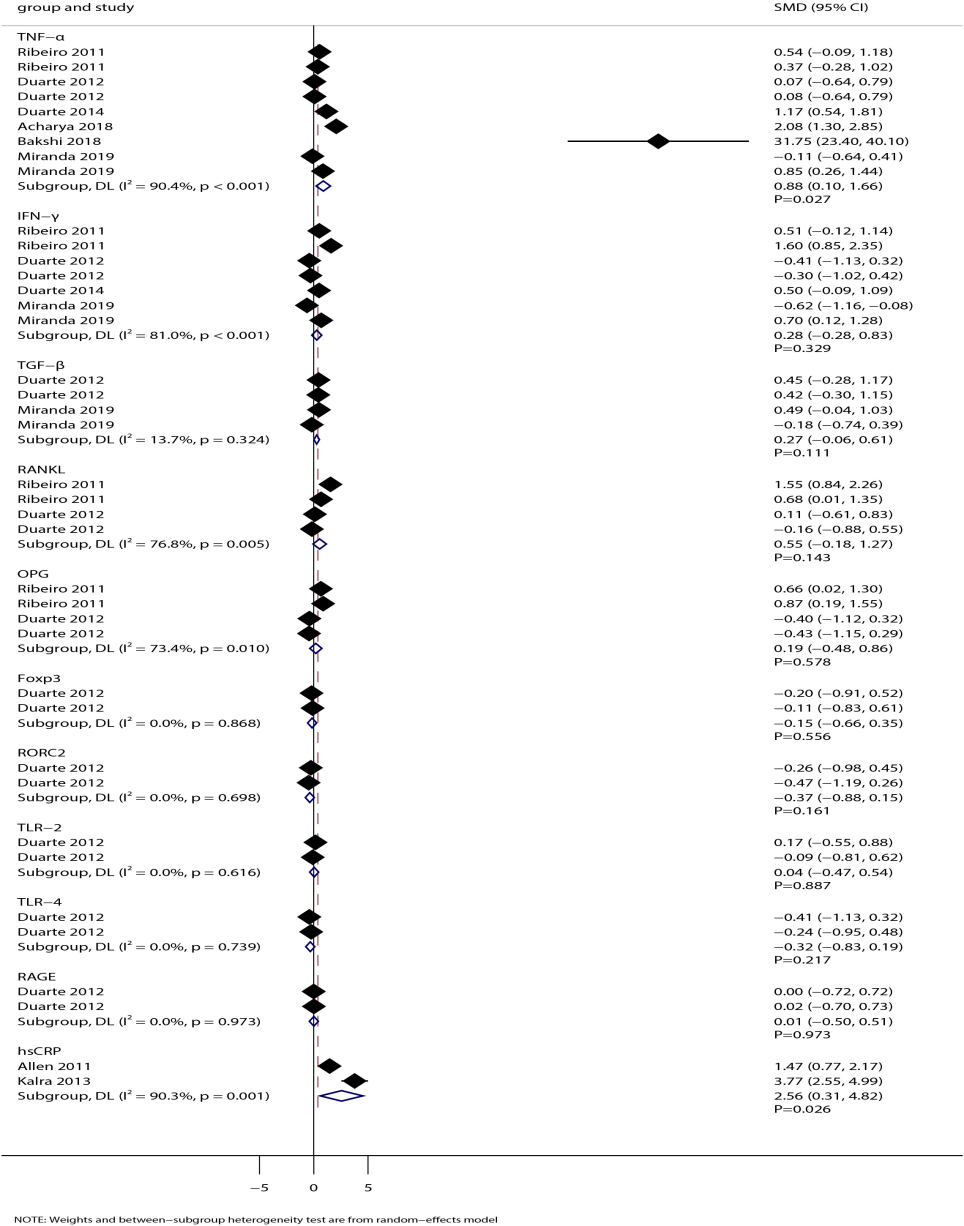
Forest plots of differences in additional inflammatory mediators between the DMCP and CP groups.

**Figure 4** presents the differences in other inflammatory mediators. The analysis showed that patients in the DMCP group were associated with higher levels of TNF-α (SMD: 0.88; 95% CI: 0.10 to 1.66; P=0.027) and high-sensitivity C-reactive protein (hs-CRP) (SMD: 2.56; 95% CI: 0.31 to 4.82; P=0.026) compared to the CP group. Conversely, no statistically significant differences were observed for interferon-gamma (IFN-γ), transforming growth factor-beta (TGF-β), receptor activator of nuclear factor-kappa B ligand (RANKL), osteoprotegerin (OPG), forkhead box P3 (Foxp3), RAR-related orphan receptor C2 (RORC2), toll-like receptor 2 (TLR-2), toll-like receptor 4 (TLR-4), or RAGE. Significant heterogeneity was detected for TNF-α (I^2^ = 90.4%; P < 0.001), IFN-γ (I^2^ = 81.0%; P < 0.001), RANKL (I^2^ = 76.8%; P=0.005), OPG (I^2^ = 73.4%; P=0.010), and hs-CRP (I^2^ = 90.3%; P=0.001), but not for TGF-β (I^2^ = 13.7%; P=0.324), Foxp3 (I^2^ = 0.0%; P=0.868), RORC2 (I^2^ = 0.0%; P=0.698), TLR-2 (I^2^ = 0.0%; P=0.616), TLR-4 (I^2^ = 0.0%; P=0.739), and RAGE (I^2^ = 0.0%; P=0.973). Sensitivity analysis indicated that the significant finding for TNF-α was unstable (**Supplementary File 2**). Furthermore, significant publication bias was identified for TNF-α (Egger’s P=0.004; Begg’s P=0.251). After adjustment, the difference in TNF-α levels was no longer statistically significant (**Supplementary File 3**).

### Oxidative stress-related biomarkers

**Figure 5:**
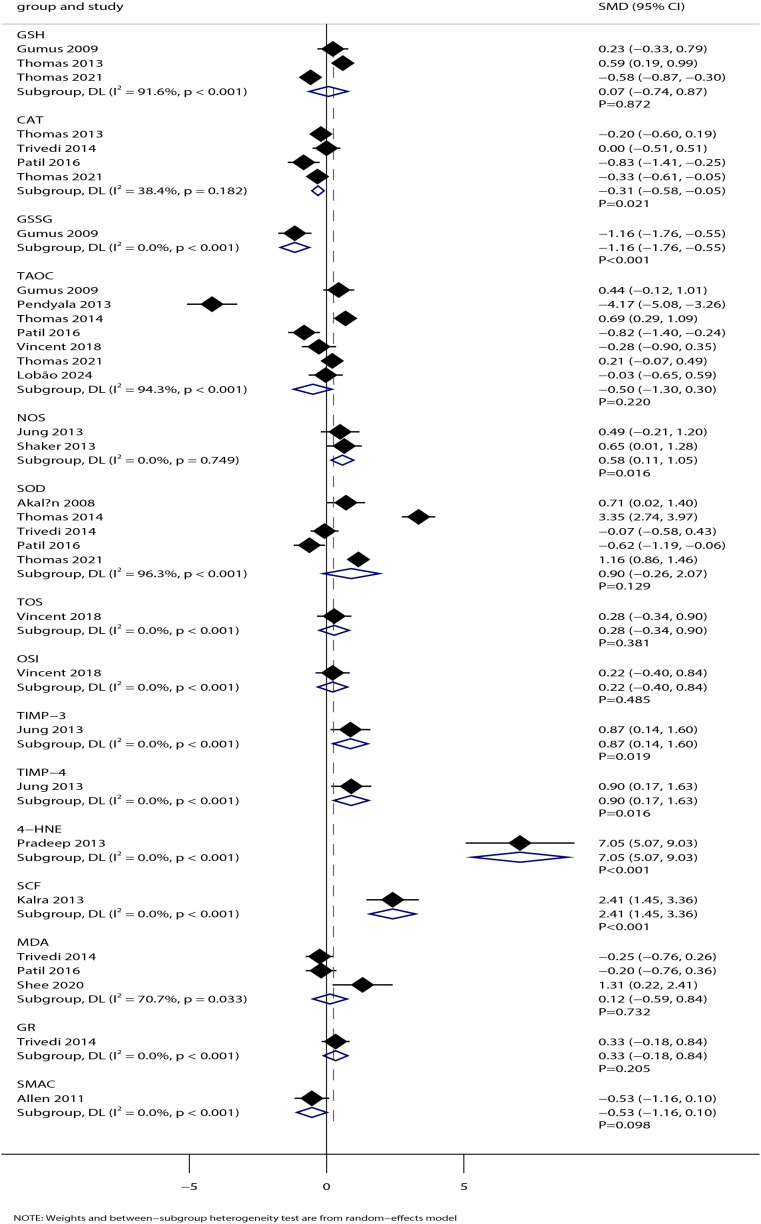
Forest plots of differences in oxidative stress-related biomarkers between the DMCP and CP groups.

**Figure 5** presents the differences in oxidative stress-related biomarkers. The analysis revealed that catalase (CAT) (SMD: -0.31; 95% CI: -0.58 to -0.05; P=0.021) and oxidized glutathione (GSSG) (SMD: -1.16; 95% CI: -1.76 to -0.55; P < 0.001) levels were significantly lower in the DMCP group. Conversely, the DMCP group were associated with higher levels of nitric oxide synthase (NOS) (SMD: 0.58; 95% CI: 0.11 to 1.05; P=0.016), tissue inhibitor of metalloproteinase-3 (TIMP-3) (SMD: 0.87; 95% CI: 0.14 to 1.60; P=0.019), tissue inhibitor of metalloproteinase-4 (TIMP-4) (SMD: 0.90; 95% CI: 0.17 to 1.63; P=0.016), 4-hydroxynonenal (4-HNE) (SMD: 7.05; 95% CI: 5.07 to 9.03; P < 0.001), and stem cell factor (SCF) (SMD: 2.41; 95% CI: 1.45 to 3.36; P < 0.001). No statistically significant differences were observed for glutathione (GSH), total antioxidant capacity (TAOC), superoxide dismutase (SOD), total oxidant status (TOS), oxidative stress index (OSI), malondialdehyde (MDA), glutathione reductase (GR), or second mitochondria-derived activator of caspase (SMAC). Significant heterogeneity was identified for GSH (I^2^ = 91.6%; P < 0.001), TAOC (I^2^ = 94.3%; P < 0.001), SOD (I^2^ = 96.3%; P < 0.001), and MDA (I^2^ = 70.7%; P=0.033), but not for CAT (I^2^ = 38.4%; P=0.182) or NOS (I^2^ = 0.0%; P=0.749). Sensitivity analysis indicated that the results for TAOC and SOD were robust (**Supplementary File 2**). Potential significant publication bias was suggested for TAOC by Begg’s test (P=0.035) but not by Egger’s test (P=0.105); the conclusion for TAOC remained unchanged after adjustment. No significant publication bias was detected for SOD (**Supplementary File 3**).

### Other biomarkers

**Figure 6:**
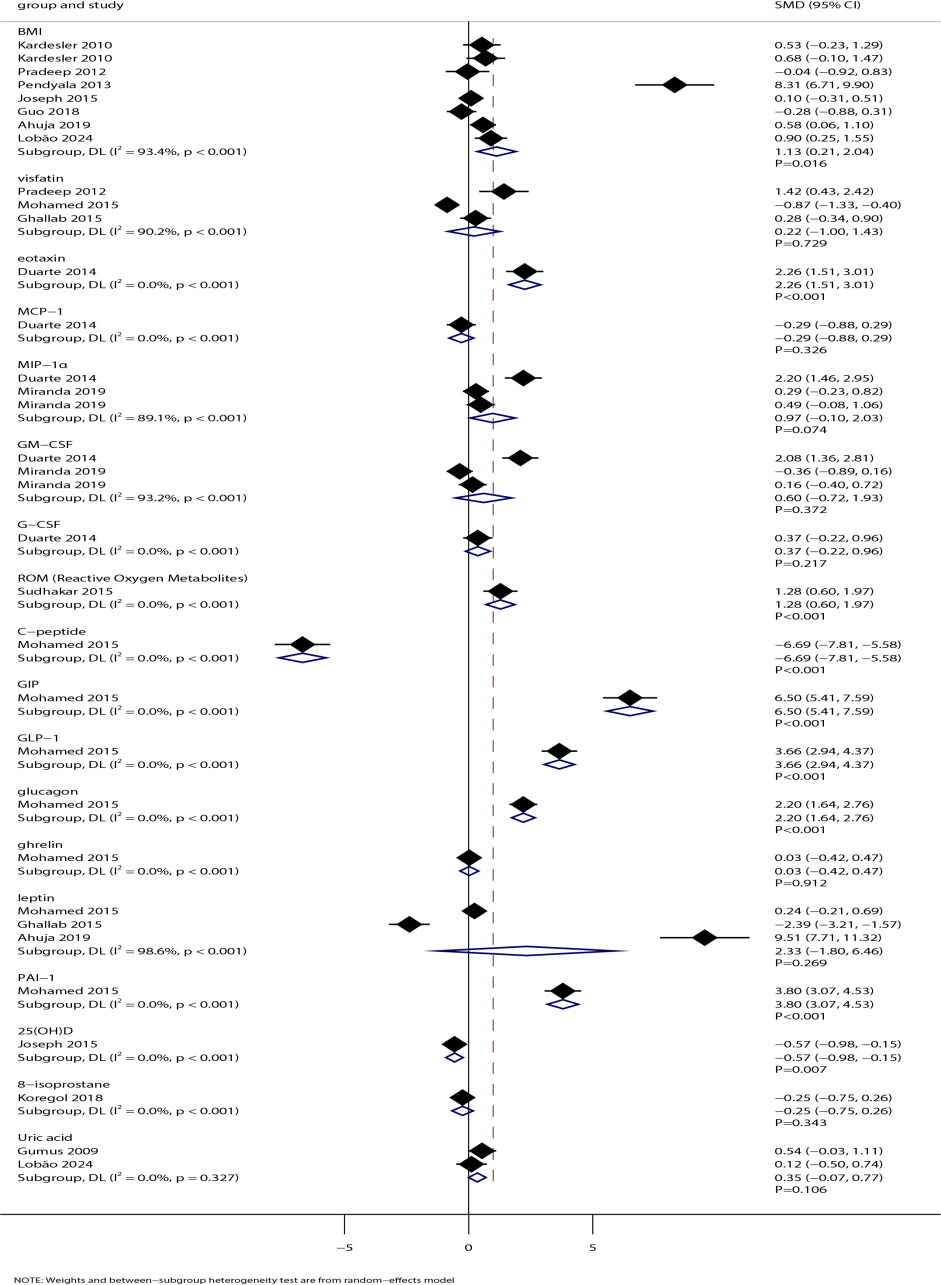
Forest plots of differences in other biomarkers between the DMCP and CP groups.

**Figure 6** shows the differences in other biomarkers. The analysis revealed that the DMCP group were associated with higher levels of body mass index (BMI) (SMD: 1.13; 95% CI: 0.21 to 2.04; P=0.016), eotaxin (SMD: 2.26; 95% CI: 1.51 to 3.01; P < 0.001), reactive oxygen metabolites (ROM) (SMD: 1.28; 95% CI: 0.60 to 1.97; P < 0.001), glucose-dependent insulinotropic polypeptide (GIP) (SMD: 6.50; 95% CI: 5.41 to 7.59; P < 0.001), glucagon-like peptide-1 (GLP-1) (SMD: 3.66; 95% CI: 2.94 to 4.37; P < 0.001), glucagon (SMD: 2.20; 95% CI: 1.64 to 2.76; P < 0.001), and plasminogen activator inhibitor-1 (PAI-1) (SMD: 3.80; 95% CI: 3.07 to 4.53; P < 0.001), along with lower levels of C-peptide (SMD: -6.69; 95% CI: -7.81 to -5.58; P < 0.001) and 25-hydroxyvitamin D [25(OH)D] (SMD: -0.57; 95% CI: -0.98 to -0.15; P=0.007). Conversely, no statistically significant intergroup differences were found for visfatin, monocyte chemoattractant protein-1 (MCP-1), macrophage inflammatory protein-1 alpha (MIP-1α), granulocyte-macrophage colony-stimulating factor (GM-CSF), granulocyte colony-stimulating factor (G-CSF), ghrelin, leptin, 8-isoprostane, or uric acid. Significant heterogeneity was observed for BMI (I^2^ = 93.4%; P < 0.001), visfatin (I^2^ = 90.2%; P < 0.001), MIP-1α (I^2^ = 89.1%; P < 0.001), GM-CSF (I^2^ = 93.2%; P < 0.001), and leptin (I^2^=98.6%; P < 0.001). Sensitivity analysis indicated that the significant difference in BMI was robust (**Supplementary File 2**). Although significant publication bias was indicated for BMI, the difference remained statistically significant after trim-and-fill adjustment (**Supplementary File 3**).

## Discussion

Through a systematic review and meta-analysis of 33 cross-sectional studies involving 1,875 participants, this study presents the first comprehensive comparison of lipid profiles, inflammatory factors, oxidative stress markers, and other biomarkers between DMCP and CP. Multiple categories of biomarkers demonstrated discriminative potential, providing evidence-based insights into pathological mechanisms and supporting the clinical differentiation between these two forms of periodontitis. Before proceeding, it is important to clarify which biomarkers demonstrate robust evidence. After rigorous sensitivity analysis and publication bias adjustment, the following biomarkers showed stable differences between DMCP and CP: VLDL, IL-8, hs-CRP, CAT, GSSG, NOS, TIMP-3, TIMP-4, 4-HNE, SCF, BMI, eotaxin, GIP, GLP-1, PAI-1, C-peptide, and 25(OH)D. In contrast, initial significant findings for TG, IL-6, and TNF-α were unstable. These three biomarkers are not recommended as discriminators in clinical practice or future research based on the current evidence.

The analysis revealed DMCP patients were associated with higher levels of VLDL, whereas no significant differences were observed in HDL, LDL, or TC. Although initial analysis also suggested elevated TG levels, sensitivity analysis indicated that this finding was unstable. Therefore, TG should not be considered a reliable discriminatory biomarker. The robust elevation of VLDL underscores the specific role of hepatic triglyceride-rich lipoprotein dysregulation in DMCP pathogenesis. Elevated TG levels were observed in conjunction with metabolic profiles characteristic of insulin resistance in diabetic patients (55). Insulin resistance is associated with reduced TG uptake and storage in adipose tissue as well as increased hepatic VLDL synthesis and release. Elevated VLDL levels correlate with lipid deposition in vascular endothelial and periodontal tissues, which may be linked to localized inflammatory activation and periodontal supporting structure destruction (56). Notably, VLDL exhibited low heterogeneity across studies, indicating consistent expression differences and suggesting its potential as a stable biomarker. In contrast, the lack of significant differences in HDL, LDL, and TC implies that conventional lipid markers may have limited utility in differentiation, possibly because they are more closely associated with systemic cardiovascular risk than localized periodontal inflammation.

Analysis of inflammatory factors revealed that patients with DMCP had significantly higher levels of IL-8, and hs-CRP than CP patients. However, contrary to our initial hypothesis, the elevations observed for IL-6 and TNF-α were not robust. Specifically, sensitivity analysis showed that the pooled effect sizes for both IL-6 and TNF-α were driven by one or a few studies, and after trim-and-fill adjustment for publication bias, the differences were no longer statistically significant. Therefore, IL-6 and TNF-α should not be considered reliable discriminatory biomarkers between DMCP and CP based on the current evidence. The elevated levels of IL-8 and hs-CRP, which remained stable across sensitivity analyses, support a “high inflammatory burden” in DMCP, but the lack of robustness for IL-6 and TNF-α suggests that the inflammatory signature of DMCP may be more complex and requires further validation. The core mechanism may be related to diabetes-induced immune dysfunction and alterations in the metabolic microenvironment (57). As a pleiotropic inflammatory cytokine, IL-6 is associated with apoptosis of periodontal ligament cells and reduced osteoblast activity; it may also correlate with insulin resistance through activation of the JAK/STAT signaling pathway, suggesting a potential link among diabetes, inflammation, and periodontal destruction (58). Elevated IL-8 levels, reflecting a potent chemokine, correlate with neutrophil infiltration into periodontal tissues, which may be associated with tissue damage via the release of proteases and reactive oxygen species (59). It is important to note that the significant findings for IL-6 and TNF-α were not robust after sensitivity analysis or adjustment for publication bias, indicating that these results should be interpreted with caution. In contrast, the lack of significant differences in other cytokines like IL-1β and IL-10 suggests their role as ‘universal’ mediators in periodontitis pathogenesis, activated in both forms and thus lacking discriminative specificity.

Analysis of oxidative stress markers demonstrated that patients with DMCP exhibited a distinct imbalance, characterized by significantly lower antioxidant capacity (e.g., CAT, GSSG) and elevated levels of pro-oxidant and tissue remodeling markers (e.g., NOS, TIMP-3, TIMP-4, 4-HNE, SCF). Notably, 4-HNE showed a remarkably high SMD, suggesting a substantial intergroup difference. These findings suggest an association between oxidative stress and DMCP. Under hyperglycemic conditions, elevated ROS generation has been observed through pathways such as the polyol pathway and AGE formation. The observed reduction in CAT activity correlates with reduced ROS clearance and is associated with lipid peroxidation and accumulation of toxic metabolites such as 4-HNE (60). As a major lipid peroxidation product, 4-HNE is associated with periodontal cell membrane damage, activation of the NF-κB pathway, and reduced osteoblast function, which may be linked to alveolar bone loss (61). The elevated expression of TIMP-3 and TIMP-4 may reflect a compensatory response to MMP overactivation in DMCP. However, this response appears insufficient to restore the MMP–TIMP balance and may correlate with tissue degradation through effects on apoptotic pathways (62). Furthermore, increased levels of SCF, a key regulator of stem cell behavior, suggest a possible association with impaired tissue regeneration in DMCP, though the exact mechanisms require further elucidation.

Among other biomarkers, DMCP patients showed significant alterations related to metabolism, immune cell recruitment, incretin function, fibrinolysis, and endocrine status (e.g., elevated BMI, eotaxin, GIP, GLP-1, PAI-1; decreased C-peptide, 25(OH)D). Elevated BMI was observed in association with obesity and DMCP; obesity correlates with systemic inflammation via adipokine secretion, which may be linked to periodontal destruction (63). Increased eotaxin levels may reflect altered immune cell recruitment in DMCP (64). Elevated levels of gut-derived incretins GIP and GLP-1 may be associated with intestinal dysfunction related to glycemic dysregulation, suggesting a potential gut–periodontium axis (65). Elevated PAI-1 levels correlate with impaired fibrinolysis and are associated with fibrin deposition, which may impede tissue repair in DMCP (66). Decreased C-peptide levels are associated with impaired β-cell function, while reduced 25(OH)D correlates with dysregulated vitamin D metabolism and immune dysfunction; both may be linked to compromised periodontal immune homeostasis (67, 68).

This study has several limitations. First, the inclusion of only cross-sectional studies precludes causal inferences between biomarker levels and disease progression. Future prospective cohort studies are needed to validate these associations. Second, and most importantly, the observed biomarker differences between DMCP and CP may be substantially confounded by glycemic status (HbA1c, diabetes duration, treatment type). Although we attempted to extract these parameters, the vast majority of included studies did not report them in a manner that would allow stratification or adjustment. Specifically, no study provided individual-level HbA1c data or performed analyses comparing well-controlled versus poorly controlled diabetic patients. Therefore, it remains entirely possible that biomarkers identified as “discriminatory” in this review—particularly inflammatory markers such as IL-6 and hs-CRP—reflect the degree of systemic hyperglycemia rather than a distinct periodontal phenotype of DMCP independent of diabetes severity. This confounding by indication is a fundamental limitation of the current evidence base and precludes any claim that these biomarkers are specific to DMCP. Third, Only 4 of 33 studies (12.1%) reported any baseline smoking status data. Among these, the reporting was inconsistent (e.g., current smokers only, ever smokers, pack-years), and none of the studies stratified biomarker analyses by smoking status or adjusted for smoking in multivariable models. With only 1–2 studies per biomarker reporting smoking data, any analysis excluding smokers or comparing smokers to non-smokers would be severely underpowered and statistically unstable. Fourth, substantial methodological heterogeneity was present across the included studies, particularly regarding biomarker sampling sources and analytical methods. The included studies utilized a range of biological samples, including gingival crevicular fluid, serum, plasma, saliva, and gingival tissue, each of which captures different aspects of the local versus systemic inflammatory burden. Additionally, assay platforms varied widely, with different sensitivity and specificity profiles. We initially planned to perform subgroup analyses stratified by sampling source or assay type to explore the sources of heterogeneity. However, for the vast majority of biomarkers identified in this review, the number of available studies was insufficient to support meaningful subgroup comparisons. Furthermore, most original studies did not report key potential confounders stratified by sampling source, precluding any reliable adjustment. Consequently, we are unable to quantitatively determine the extent to which sampling source or measurement method contributed to the observed heterogeneity. The lack of subgroup analyses to explore these sources is another limitation. Therefore, future multi-center, large-scale prospective studies with rigorous confounding control are recommended to clarify the dynamic changes and prognostic relevance of these biomarkers.

## Conclusions

This systematic review and meta-analysis suggests distinct expression patterns of lipid metabolites, inflammatory mediators, oxidative stress markers, and other biomarkers in DMCP compared to CP. After rigorous sensitivity analysis and adjustment for publication bias, biomarkers demonstrating relatively robust differences include VLDL, IL-8, hs-CRP, CAT, GSSG, NOS, TIMP-3, TIMP-4, 4-HNE, SCF, eotaxin, GIP, GLP-1, PAI-1, BMI, C-peptide, and 25(OH)D. In contrast, initial findings for TG, IL-6, and TNF-α were unstable and, for IL-6 and TNF-α, became non-significant after bias adjustment; these should not be considered reliable discriminators. The robust biomarkers are closely tied to diabetes-driven metabolic dysregulation, inflammatory activation, and oxidative stress imbalance. However, due to the cross-sectional design of included studies, lack of adjustment for glycemic control, and substantial methodological heterogeneity, these findings should be considered hypothesis-generating rather than conclusive. They provide an evidence-based foundation for future prospective studies and should not yet be translated into clinical practice without independent validation.

## Data Availability

The original contributions presented in the study are included in the article/**Supplementary Material**, further inquiries can be directed to the corresponding author/s.
