# Supplementary material for: Biomarkers for differentiating diabetic periodontitis from chronic periodontitis: a systematic review and meta-analysis
Source: Front Immunol. 2026 Jun 10;17:1758079. doi: 10.3389/fimmu.2026.1758079 (PMC13290632; doi:10.3389/fimmu.2026.1758079)
Supplement: Supplementary file 1 [file Table1.docx]

PubMed: (("diabetic periodontitis"[MeSH Terms] OR "diabetic periodontitis"[Title/Abstract] OR "diabetes mellitus complicated with periodontitis"[Title/Abstract] OR "diabetes complicated with periodontitis"[Title/Abstract]) AND ("chronic periodontitis"[MeSH Terms] OR "chronic periodontitis"[Title/Abstract])) AND (biomarkers[MeSH Terms] OR "biological markers"[MeSH Terms] OR "inflammatory factors"[Title/Abstract] OR "matrix metalloproteinases"[MeSH Terms] OR "advanced glycation end products"[MeSH Terms])

EmBase: ('diabetic periodontitis'/exp OR 'diabetes mellitus complicated with periodontitis' OR 'diabetes complicated with periodontitis') AND ('chronic periodontitis'/exp OR 'chronic periodontitis') AND ('biomarker'/exp OR 'biological marker' OR 'inflammatory factor' OR 'matrix metalloproteinase'/exp OR 'advanced glycation end product'/exp)

Cochrane library: (diabetic periodontitis OR "diabetes mellitus complicated with periodontitis" OR "diabetes complicated with periodontitis") AND "chronic periodontitis" AND (biomarkers OR "biological markers" OR "inflammatory factors" OR "matrix metalloproteinases" OR "advanced glycation end products")

Web of Science: TI=(diabetic periodontitis OR "diabetes mellitus complicated with periodontitis" OR "diabetes complicated with periodontitis") AND TI=(chronic periodontitis) AND TI=(biomarkers OR "biological markers" OR "inflammatory factors" OR "matrix metalloproteinases" OR "advanced glycation end products") OR AB=(diabetic periodontitis OR "diabetes mellitus complicated with periodontitis" OR "diabetes complicated with periodontitis") AND AB=(chronic periodontitis) AND AB=(biomarkers OR "biological markers" OR "inflammatory factors" OR "matrix metalloproteinases" OR "advanced glycation end products")
