# Supplementary material for: Biomarkers for differentiating diabetic periodontitis from chronic periodontitis: a systematic review and meta-analysis
Source: Front Immunol. 2026 Jun 10;17:1758079. doi: 10.3389/fimmu.2026.1758079 (PMC13290632; doi:10.3389/fimmu.2026.1758079)
Supplement: Supplementary file 2 [file Table2.docx]

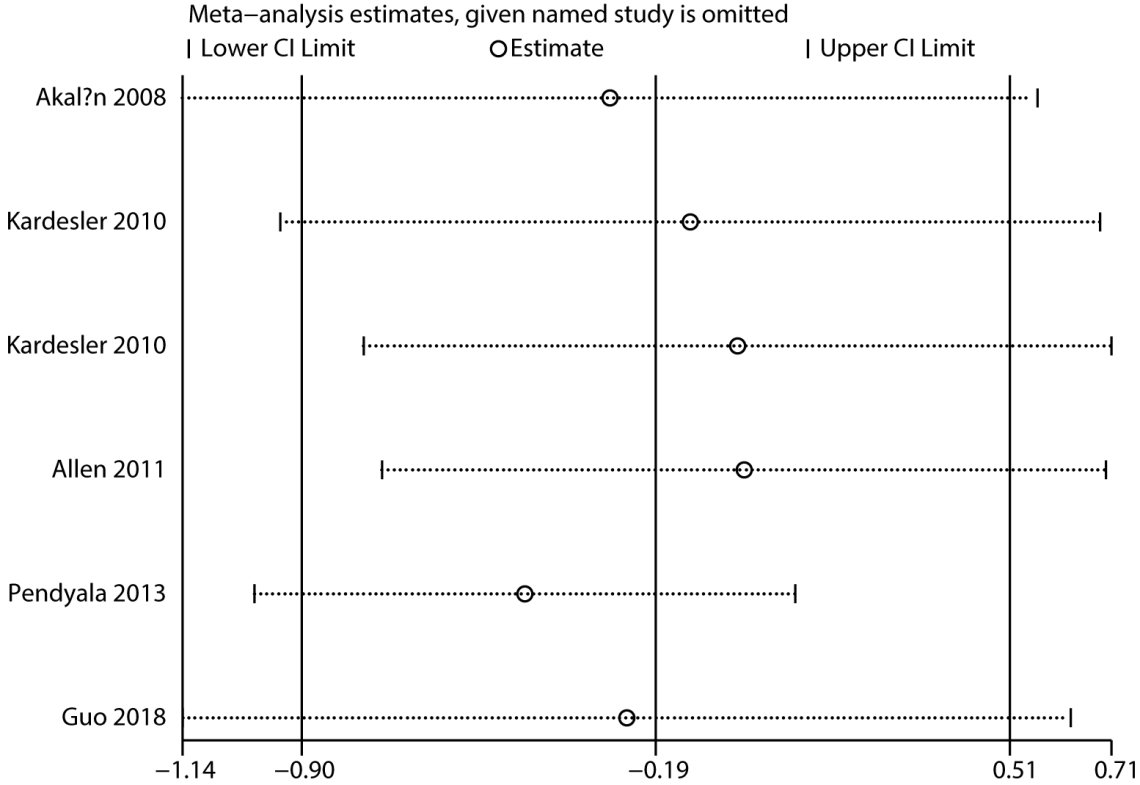


Figure S1. Sensitivity for HDL


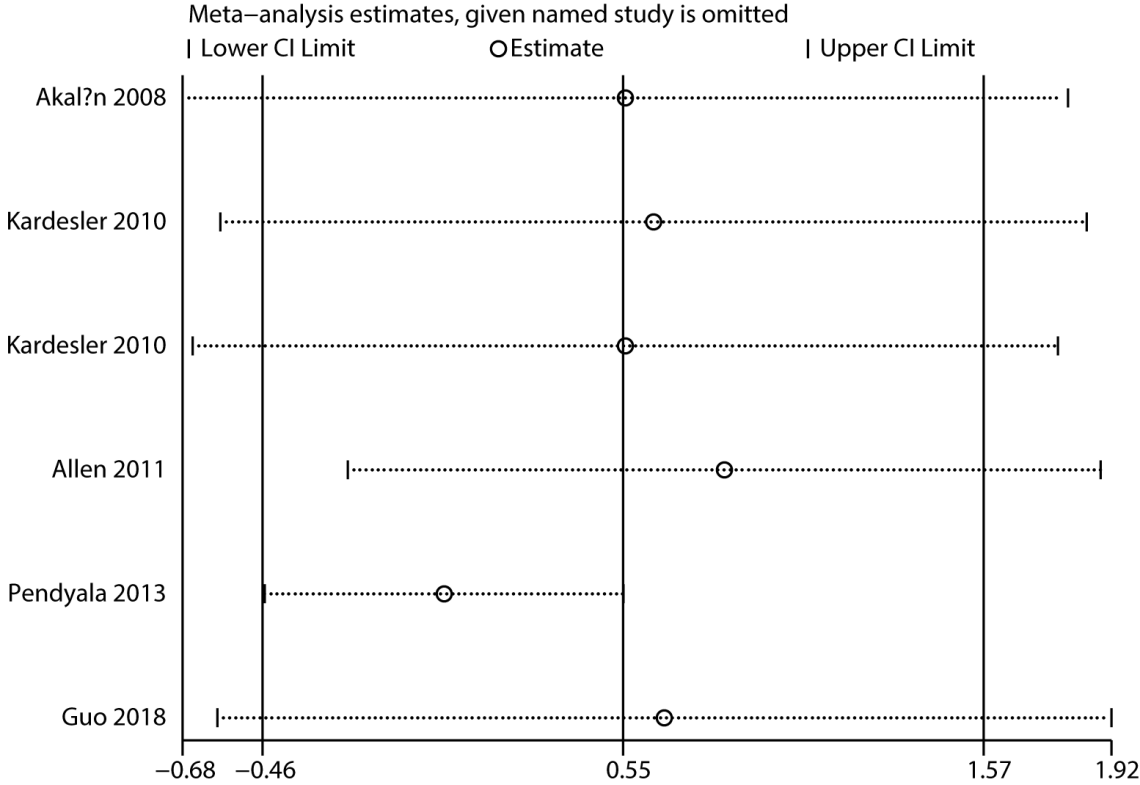


Figure S2. Sensitivity for LDL


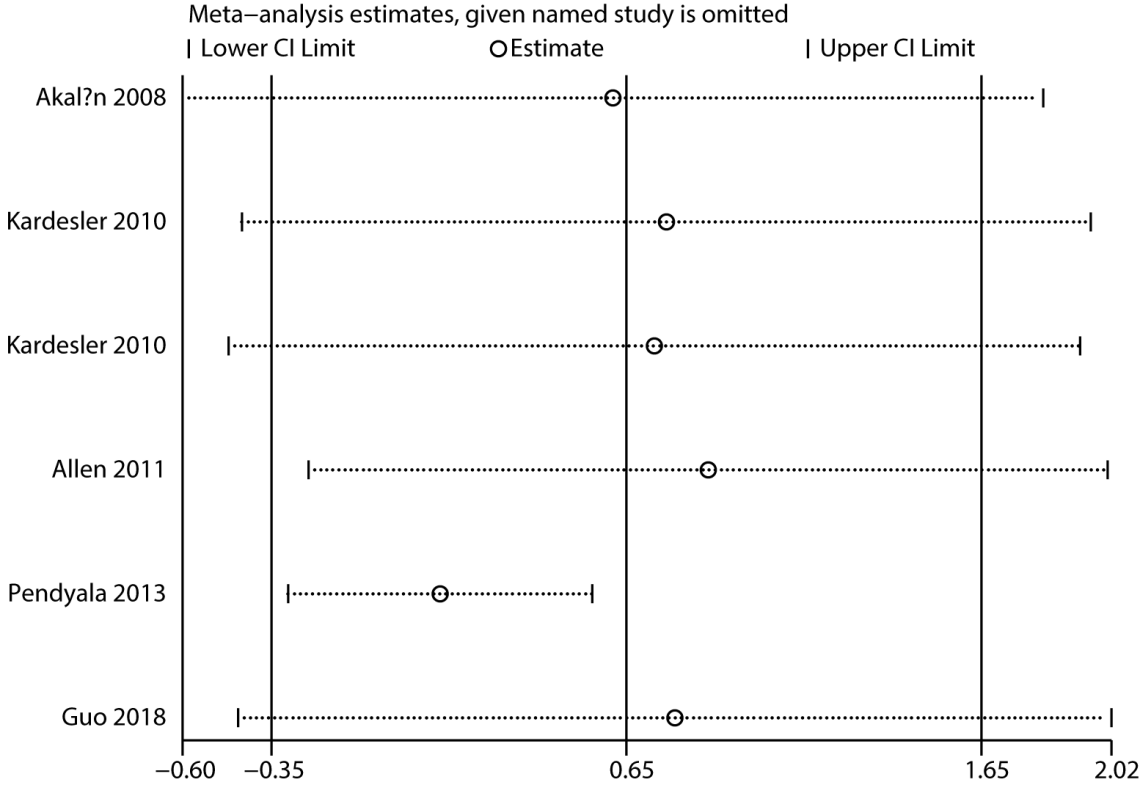


Figure S3. Sensitivity for TC


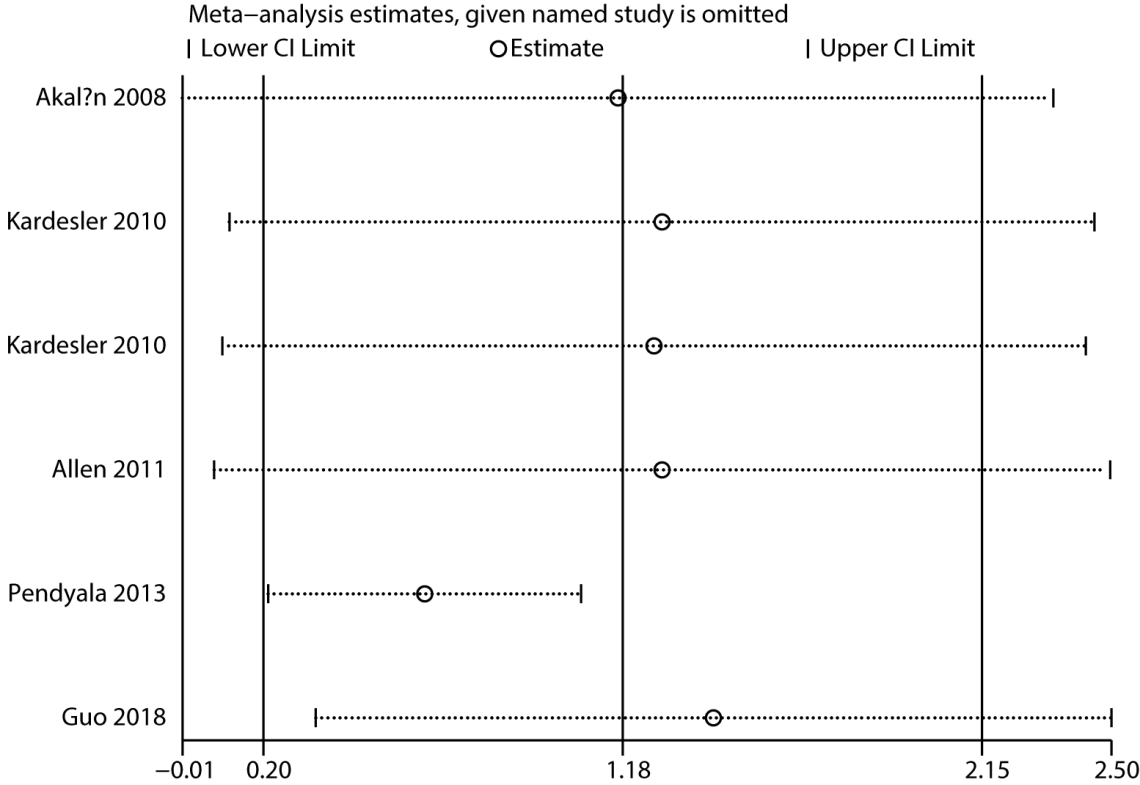


Figure S4. Sensitivity for TG


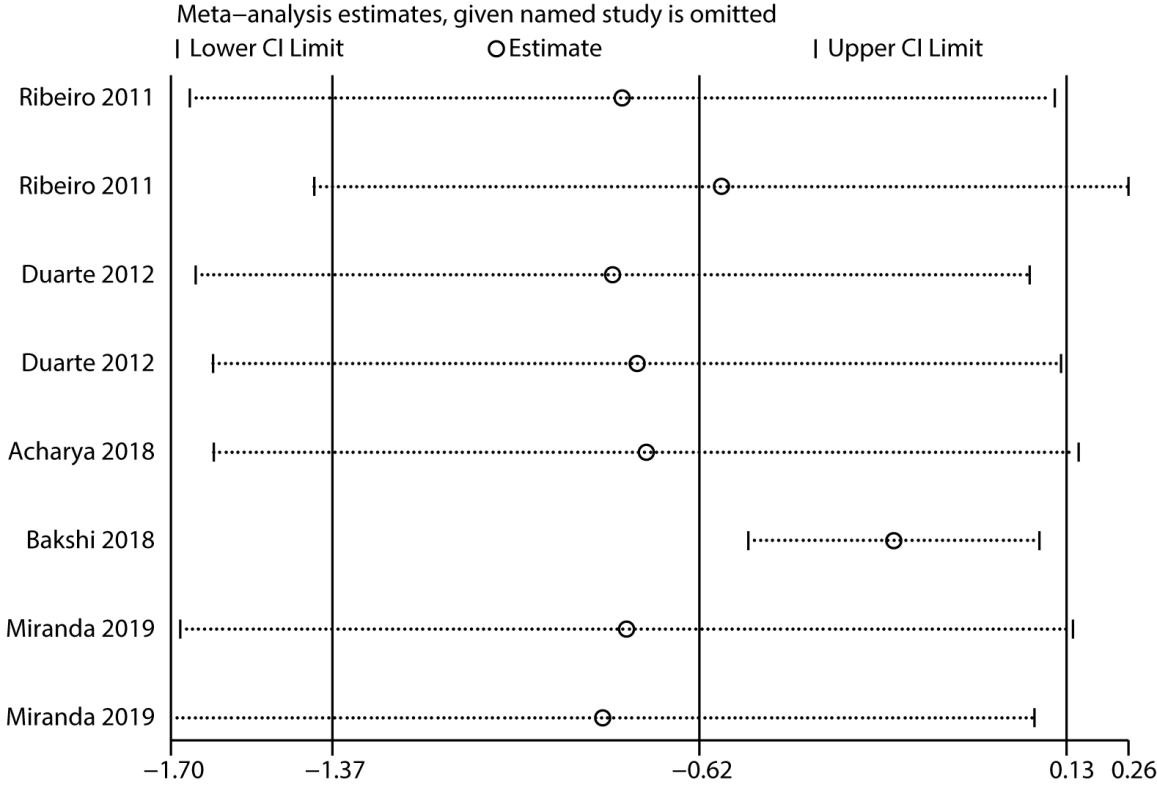


Figure S5. Sensitivity for IL-4


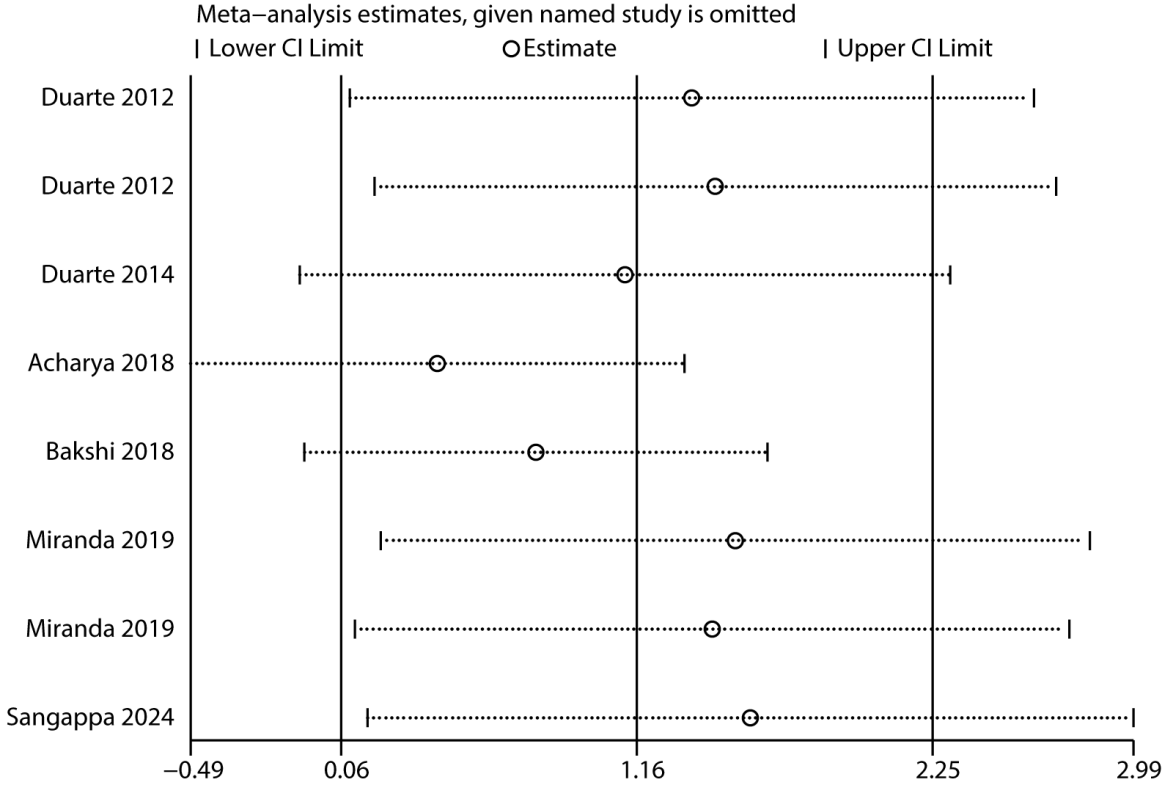


Figure S6. Sensitivity for IL-6


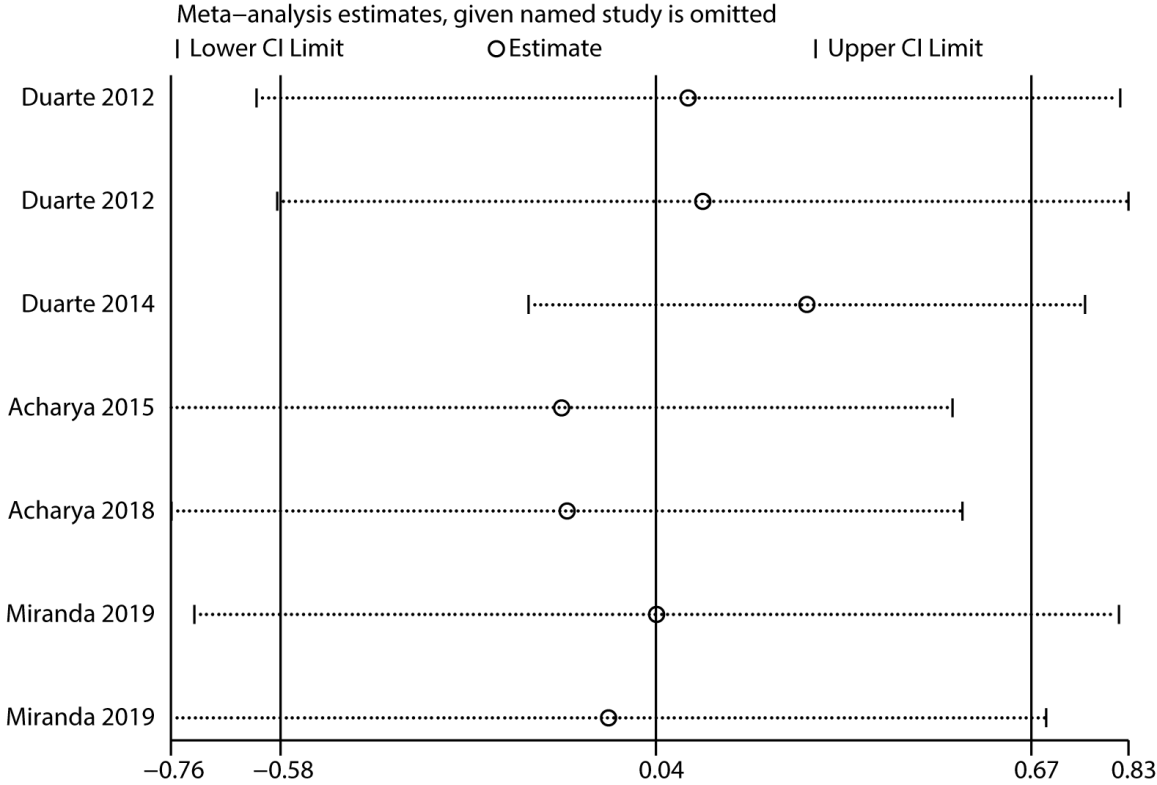


Figure S7. Sensitivity for IL-10


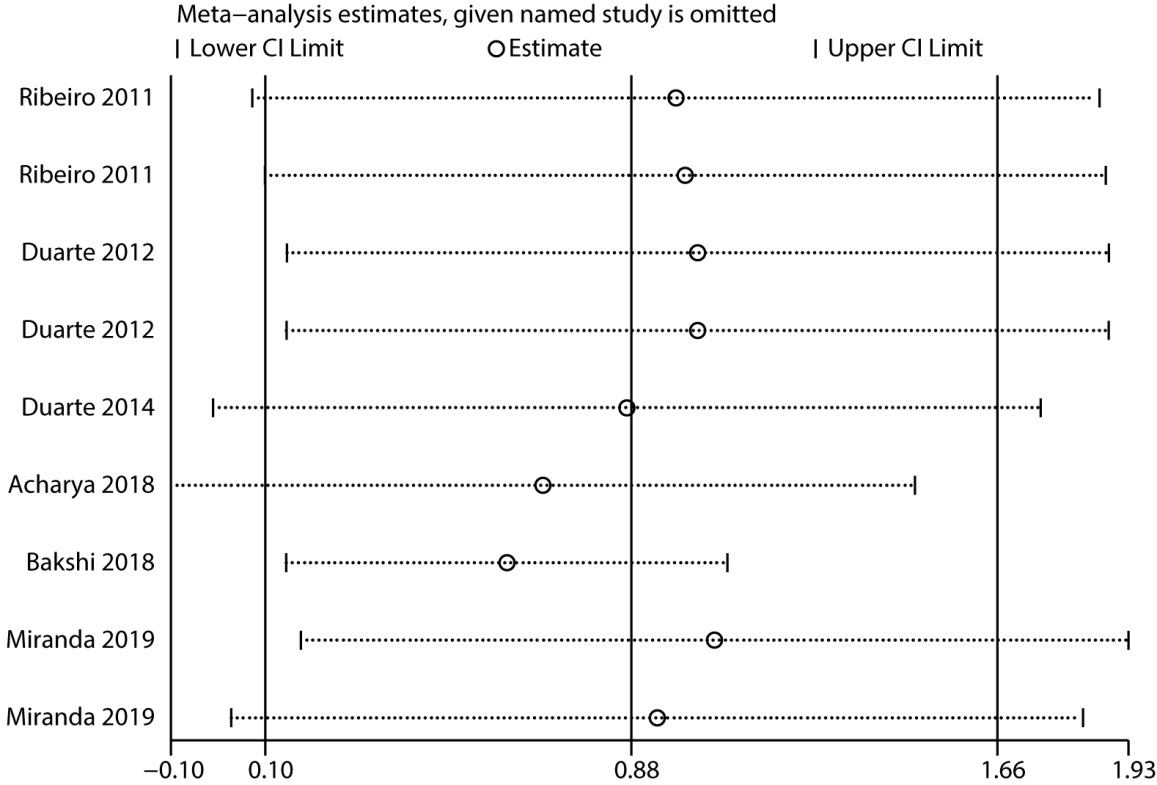


Figure S8. Sensitivity for TNF-α


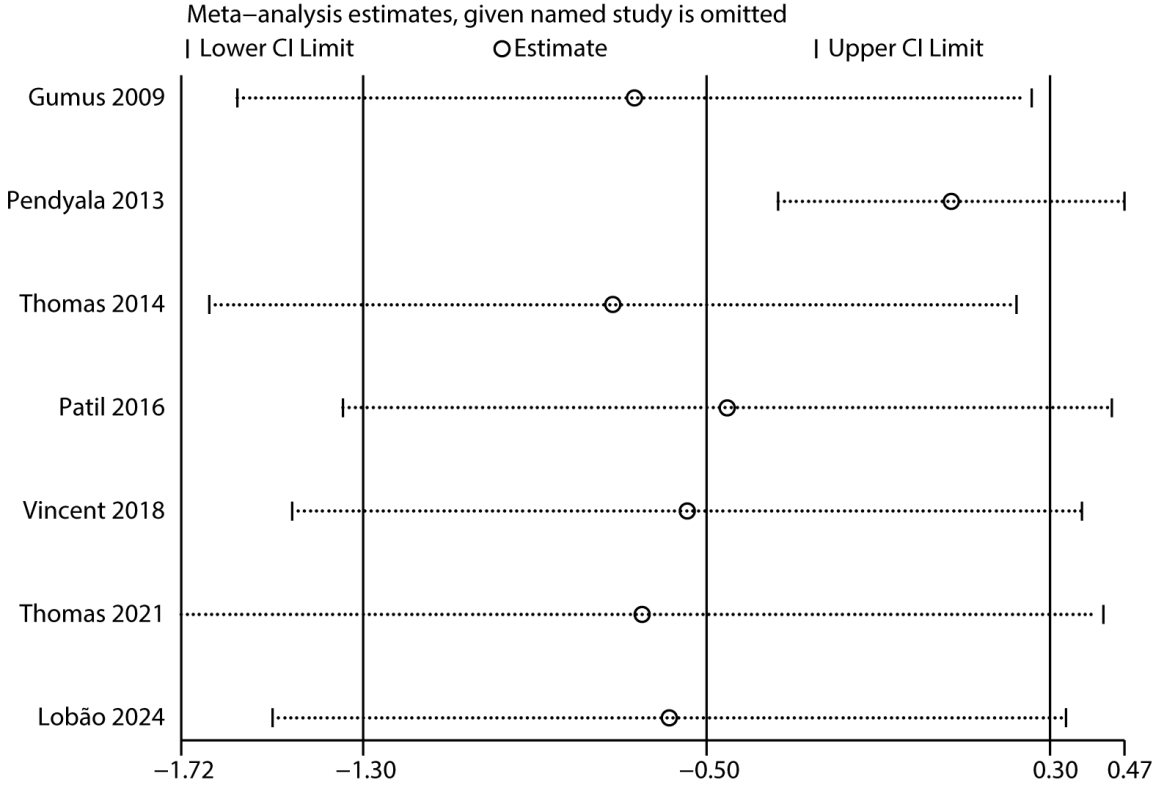


Figure S9. Sensitivity for TAOC


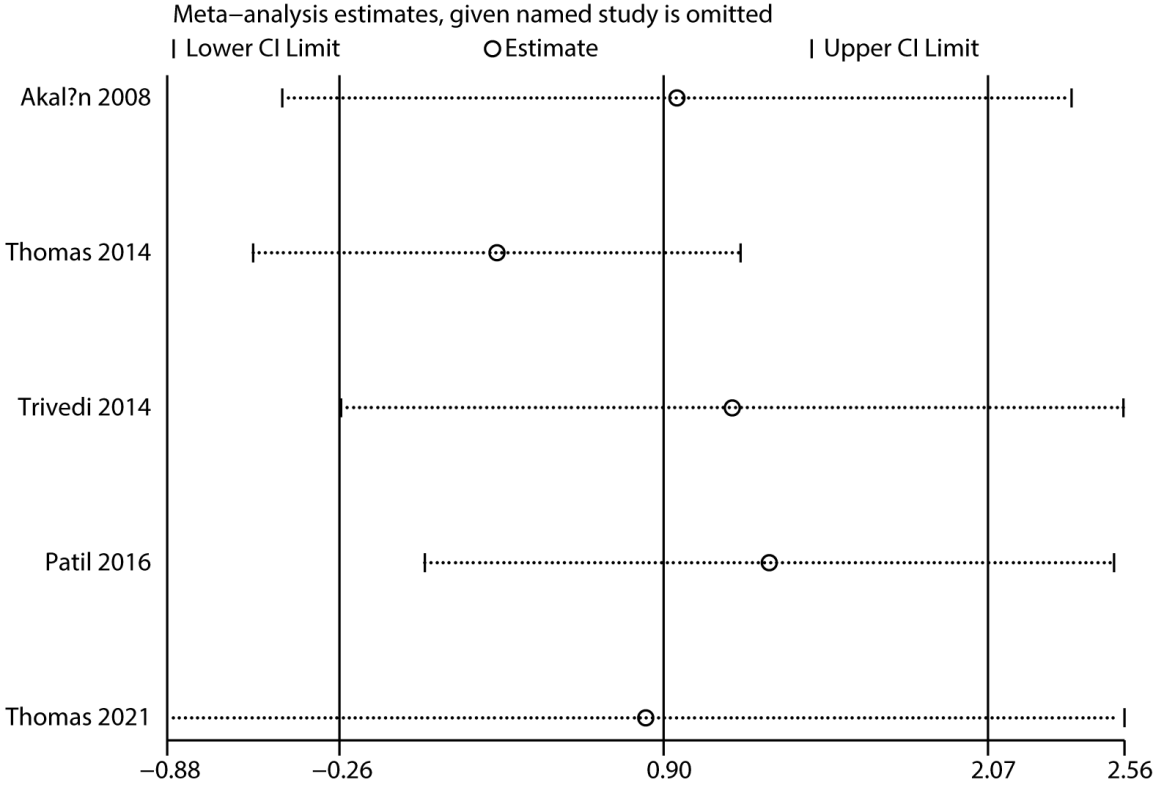


Figure S10. Sensitivity for SOD


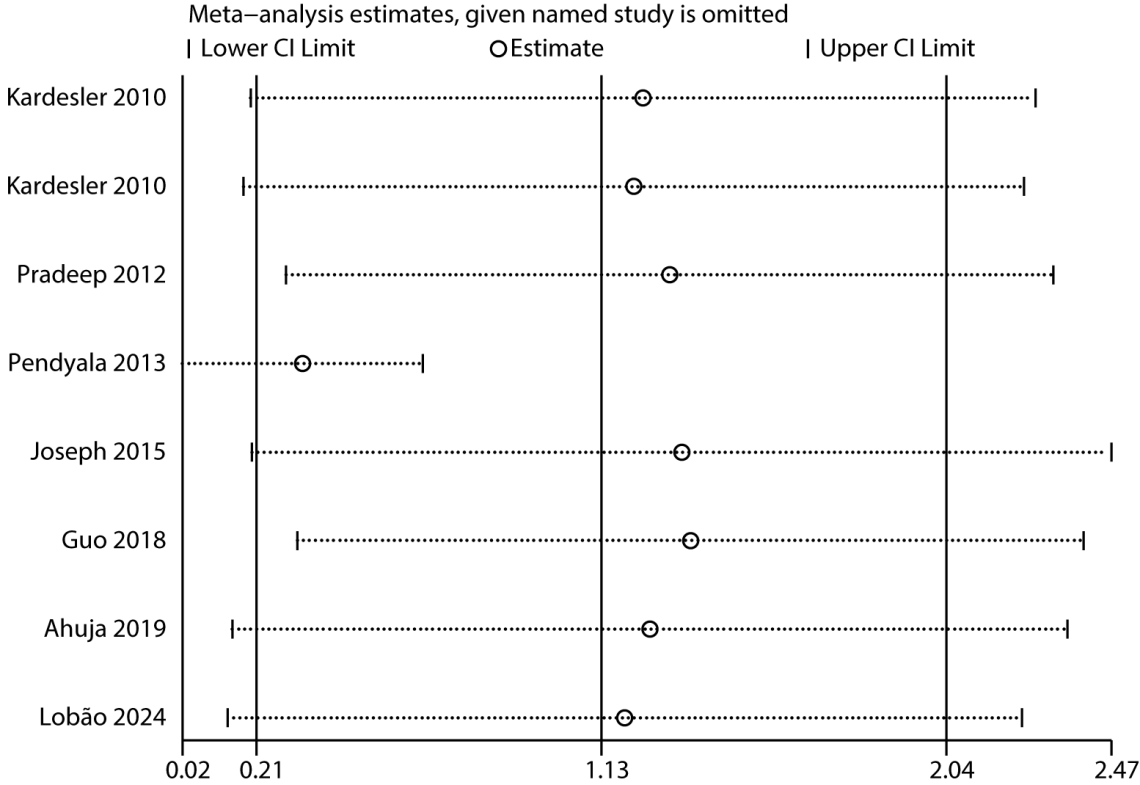


Figure S11. Sensitivity for BMI
