# Supplementary material for: Biomarkers for differentiating diabetic periodontitis from chronic periodontitis: a systematic review and meta-analysis
Source: Front Immunol. 2026 Jun 10;17:1758079. doi: 10.3389/fimmu.2026.1758079 (PMC13290632; doi:10.3389/fimmu.2026.1758079)
Supplement: Supplementary file 3 [file Table3.docx]

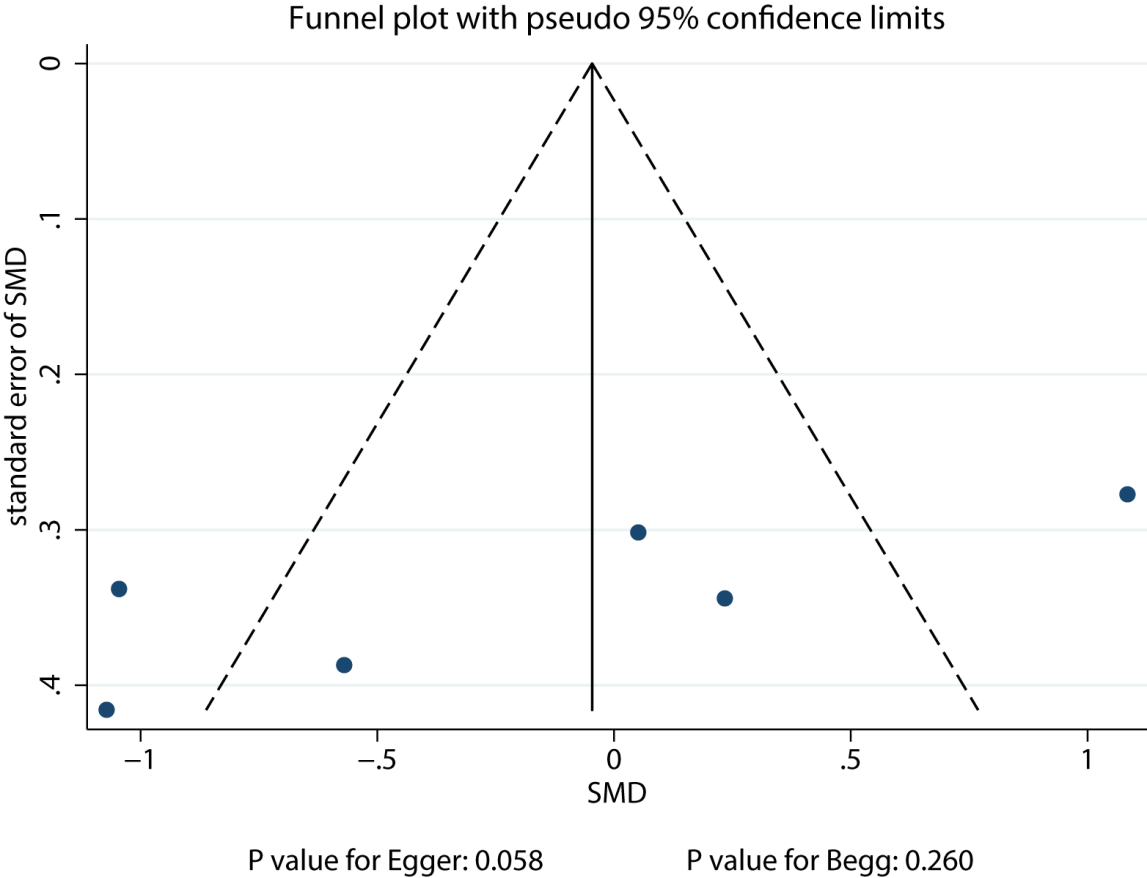


Figure S1. Funnel plot for HDL


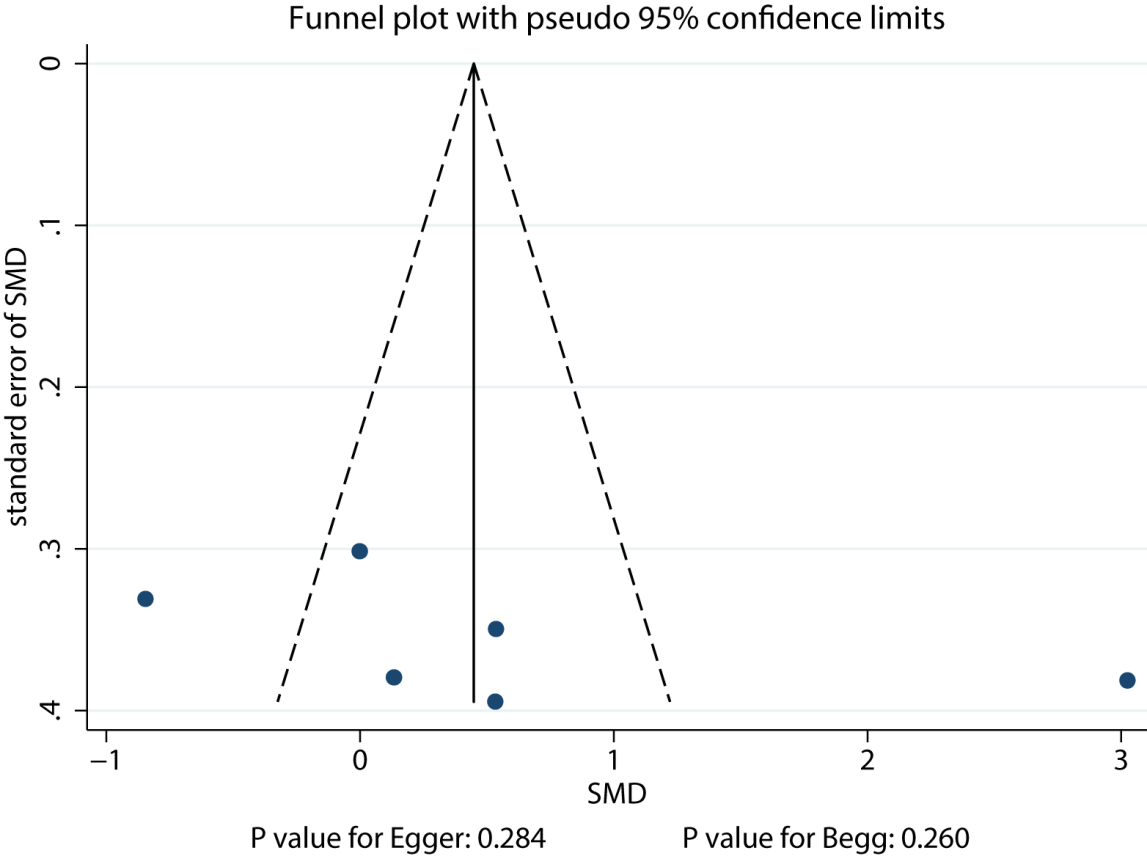


Figure S2. Funnel plot for LDL


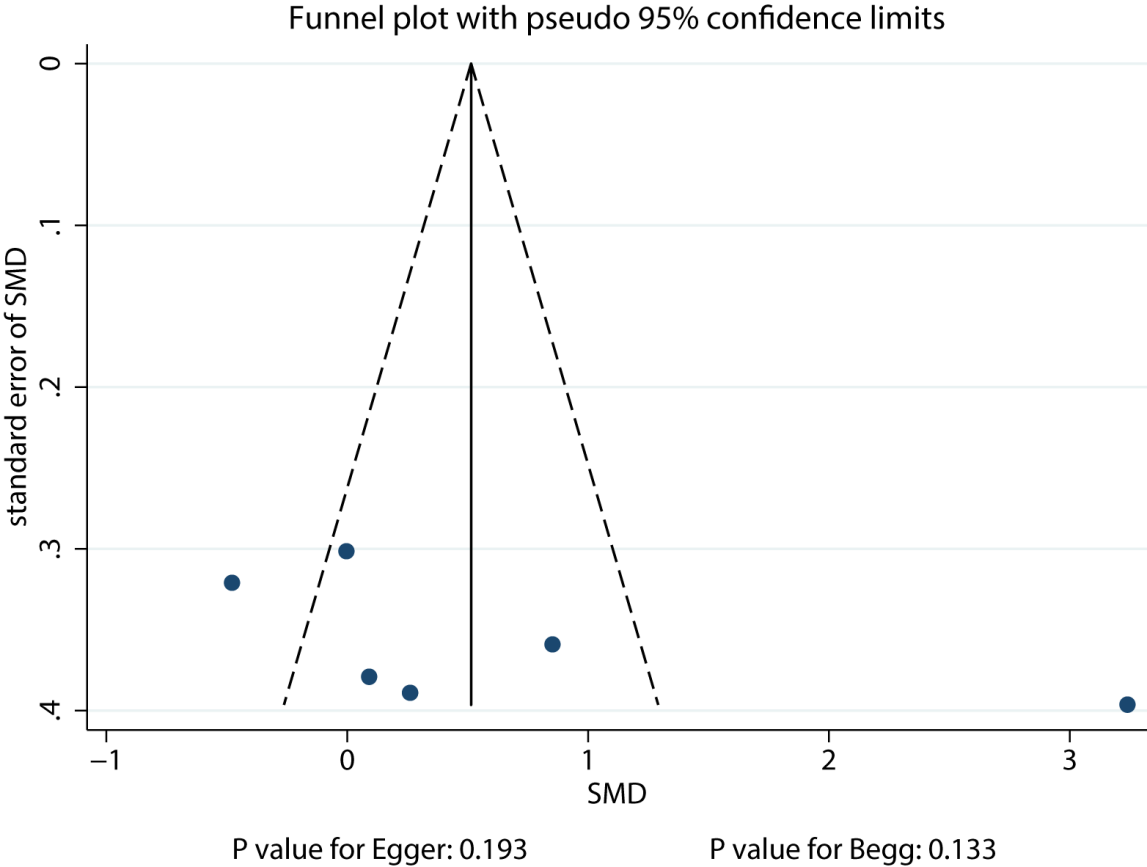


Figure S3. Funnel plot for TC


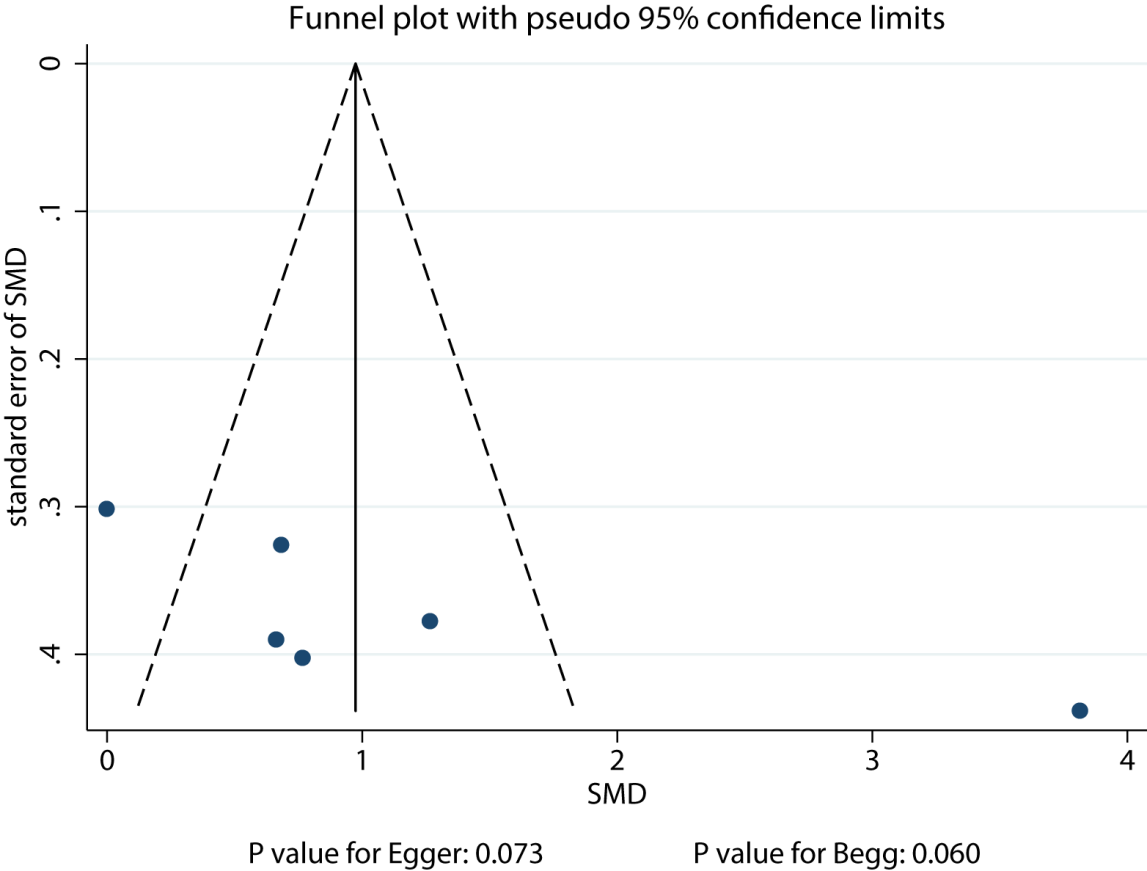


Figure S4. Funnel plot for TG


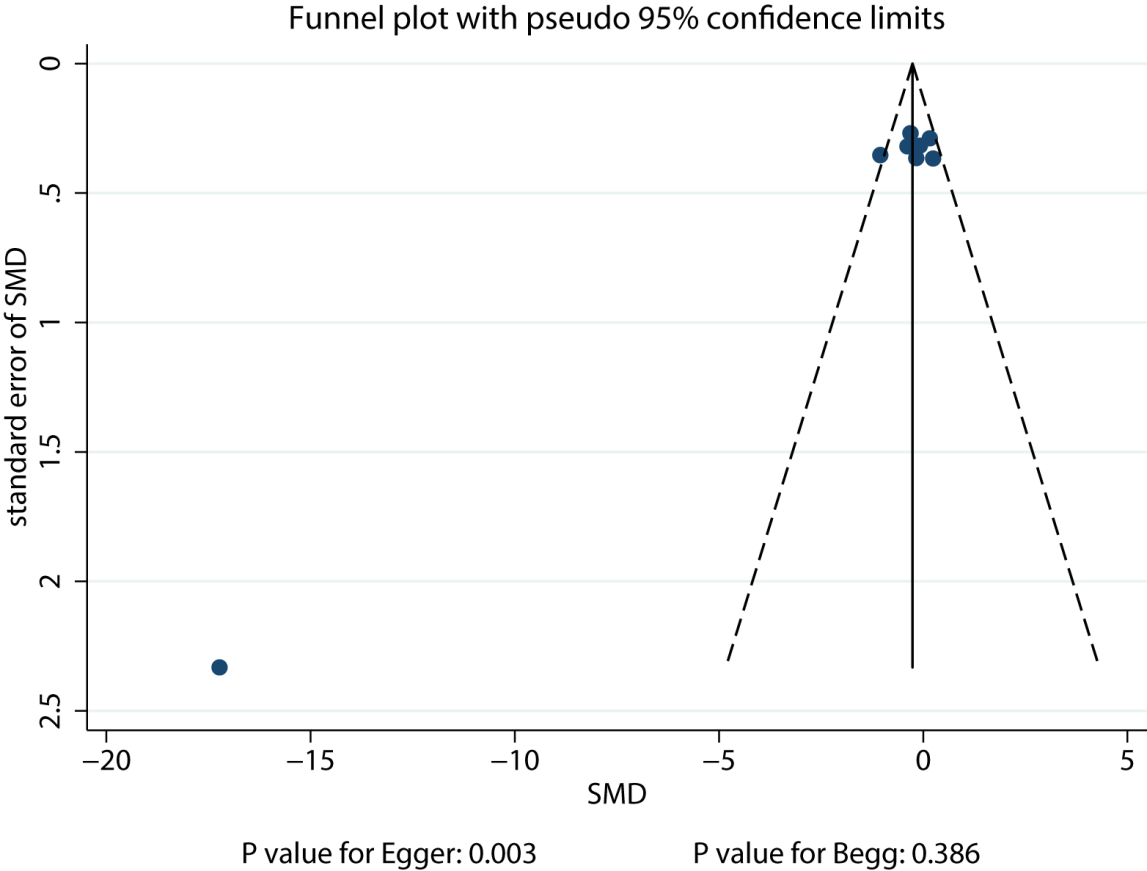


Figure S5. Funnel plot for IL-4


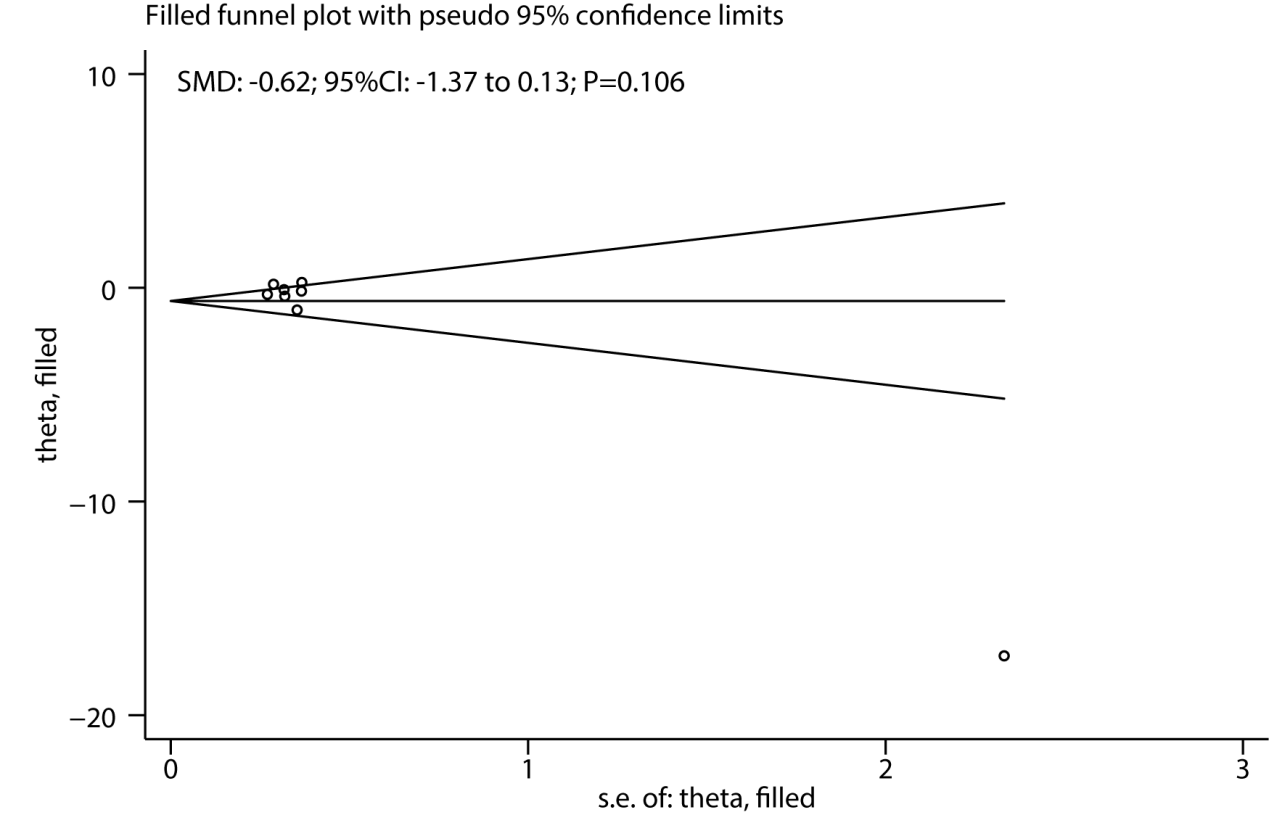


Figure S6. Trim and fill for IL-4


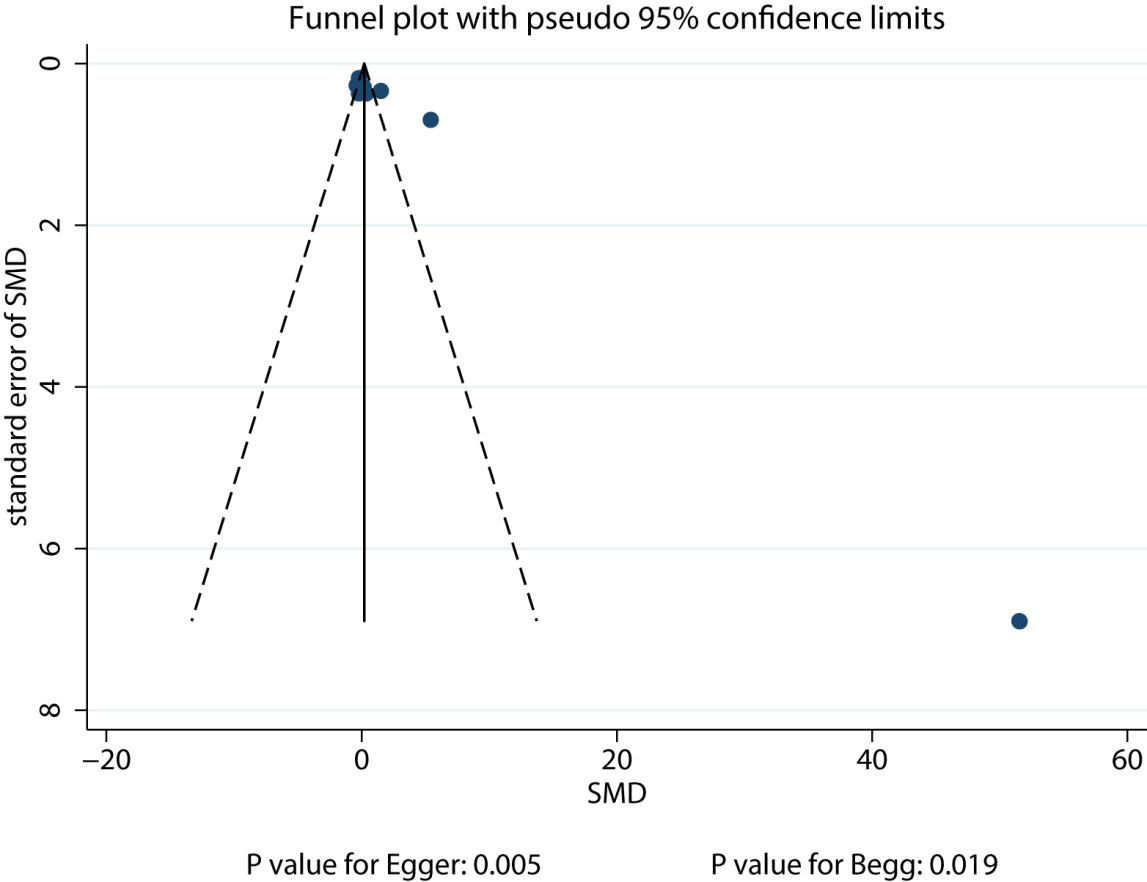


Figure S7. Funnel plot for IL-6


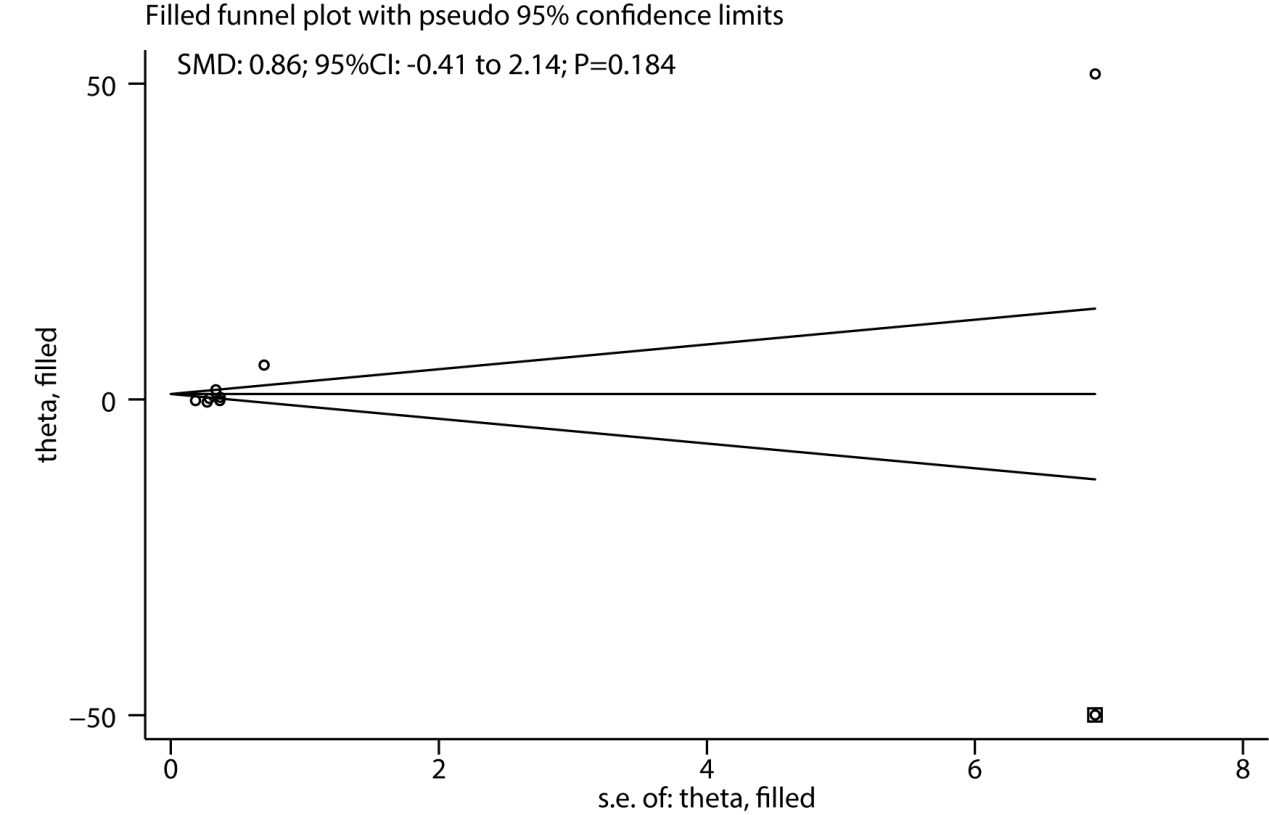


Figure S8. Trim and fill for IL-6


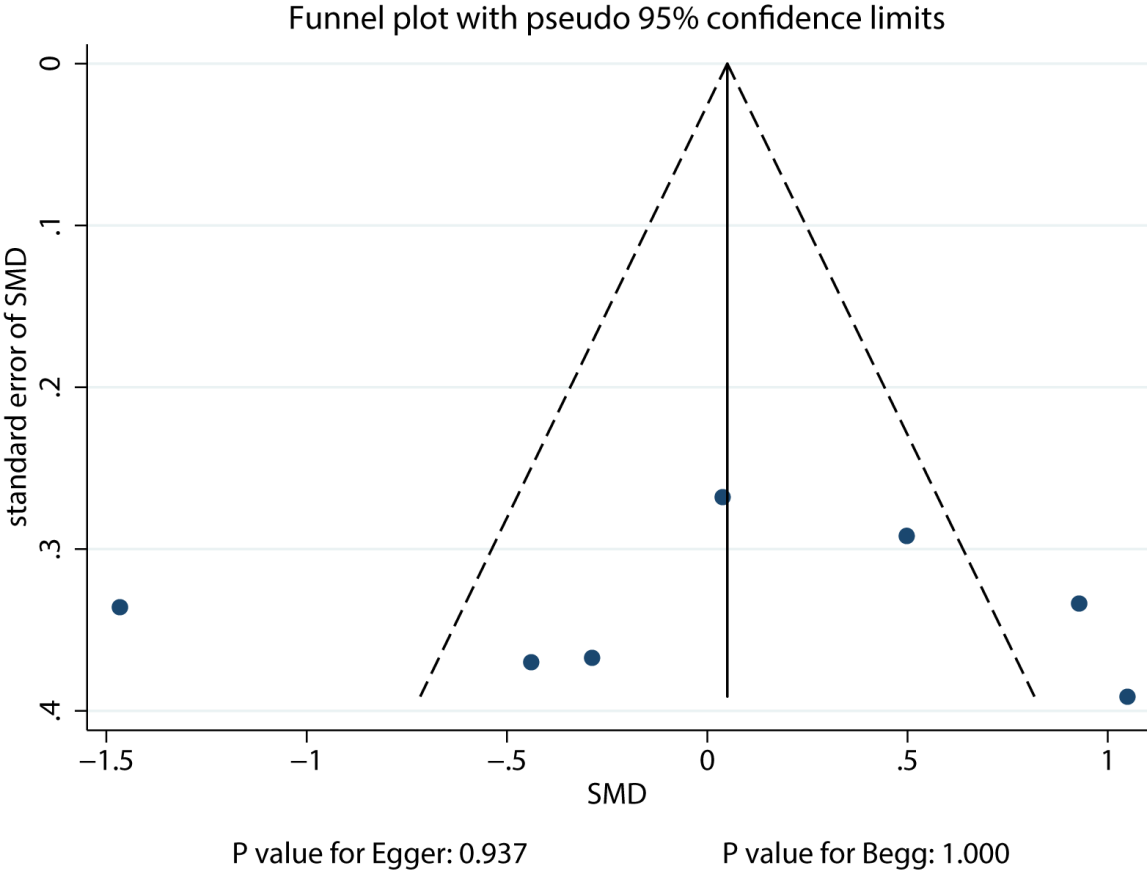


Figure S9. Funnel plot for IL-10


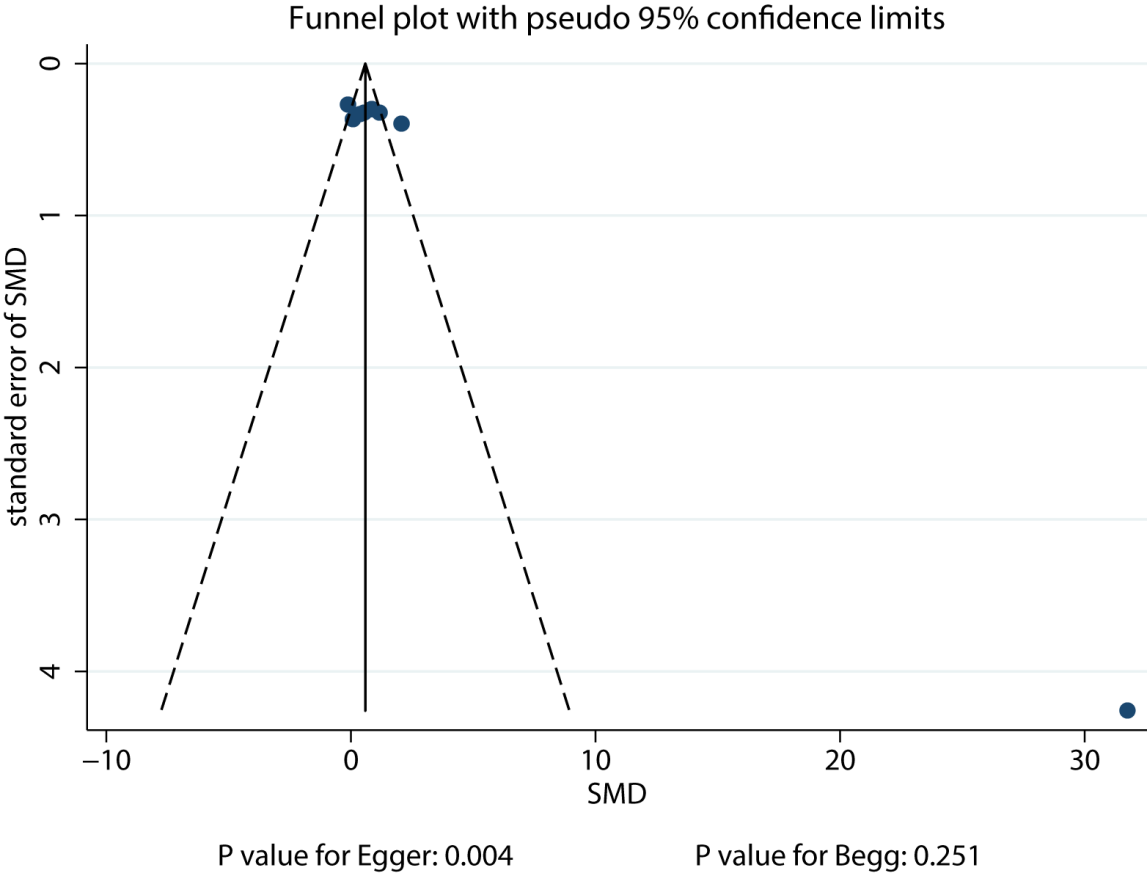


Figure S10. Funnel plot for TNF-α


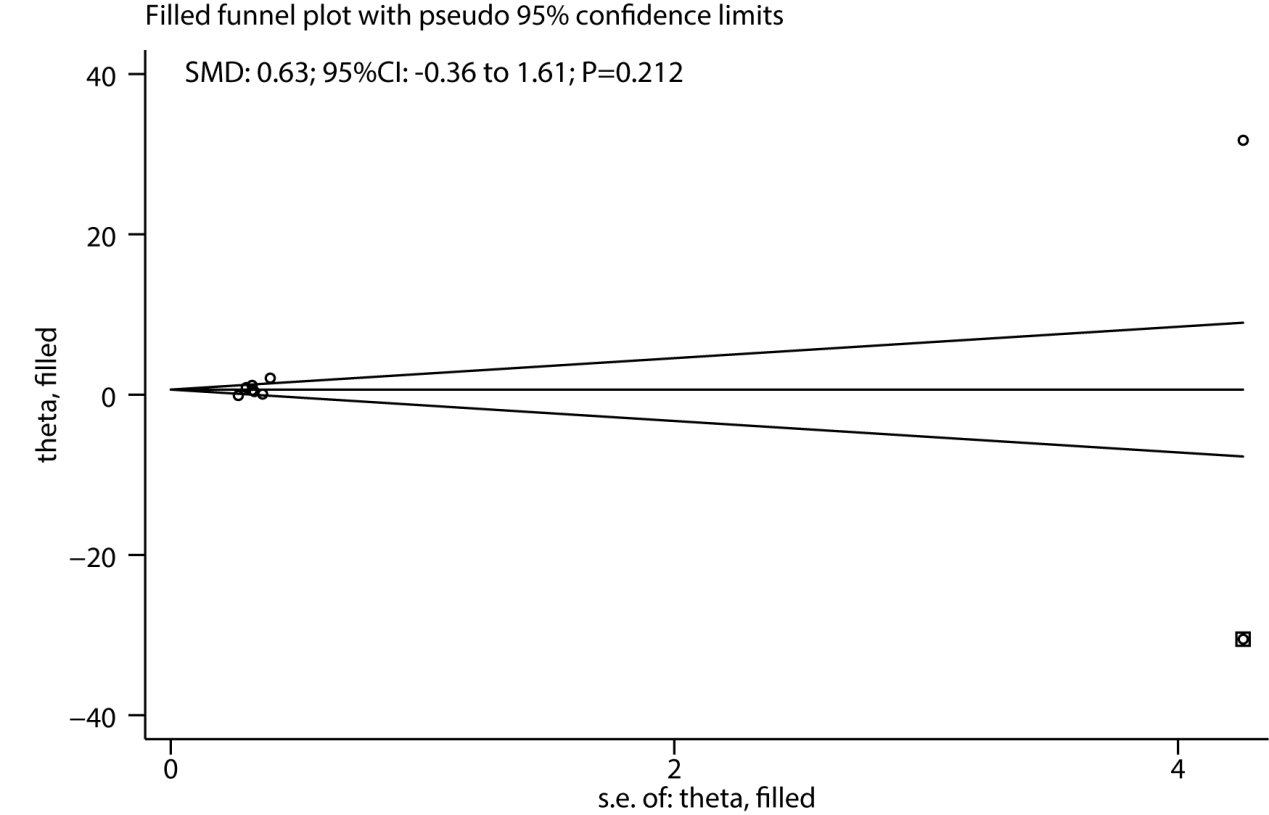


Figure S11. Trim and fill for TNF-α


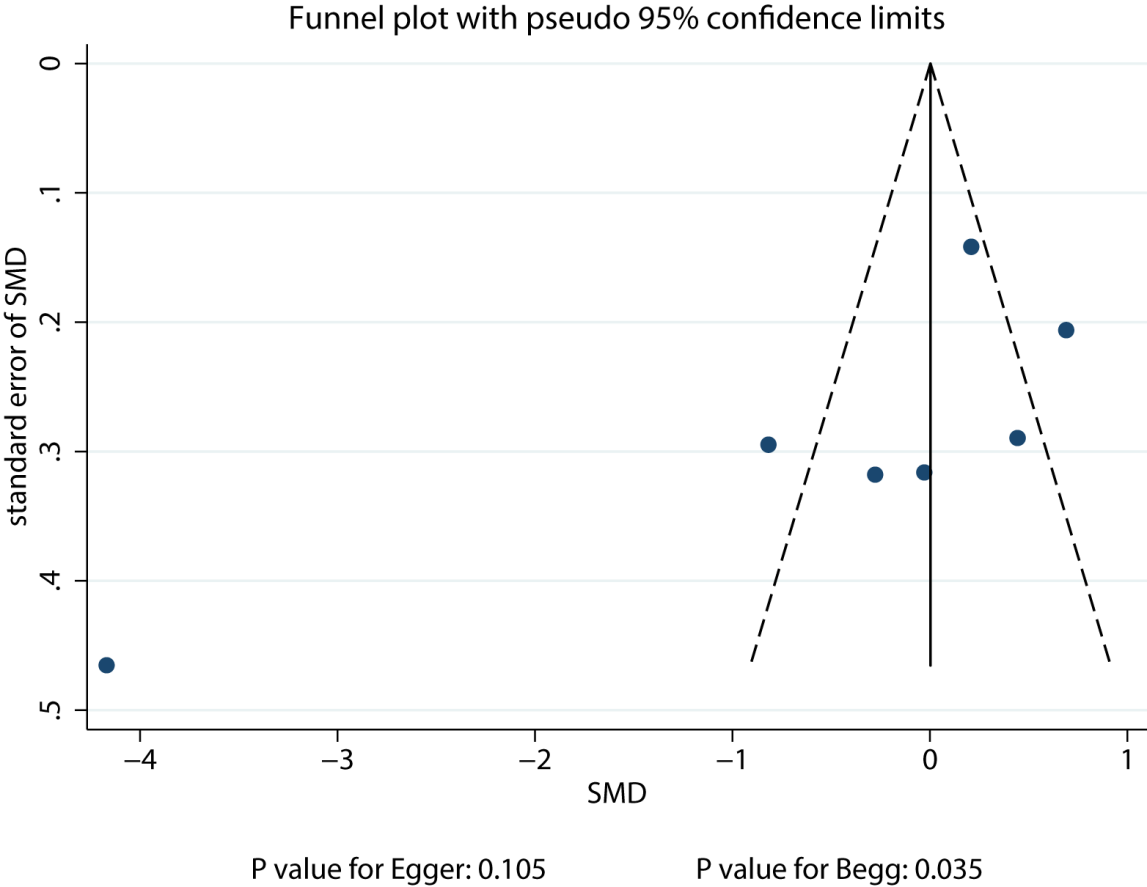


Figure S12. Funnel plot for TAOC


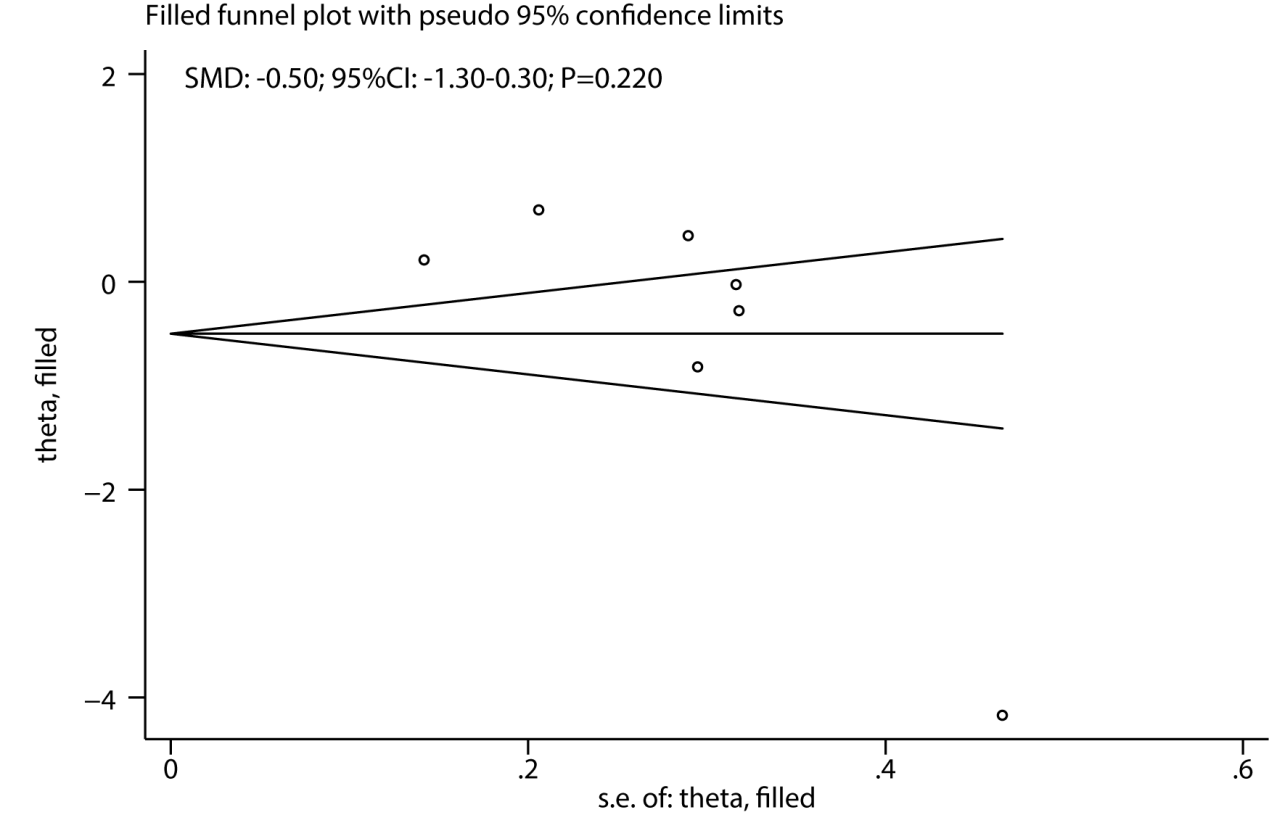


Figure S13. Trim and fill for TAOC


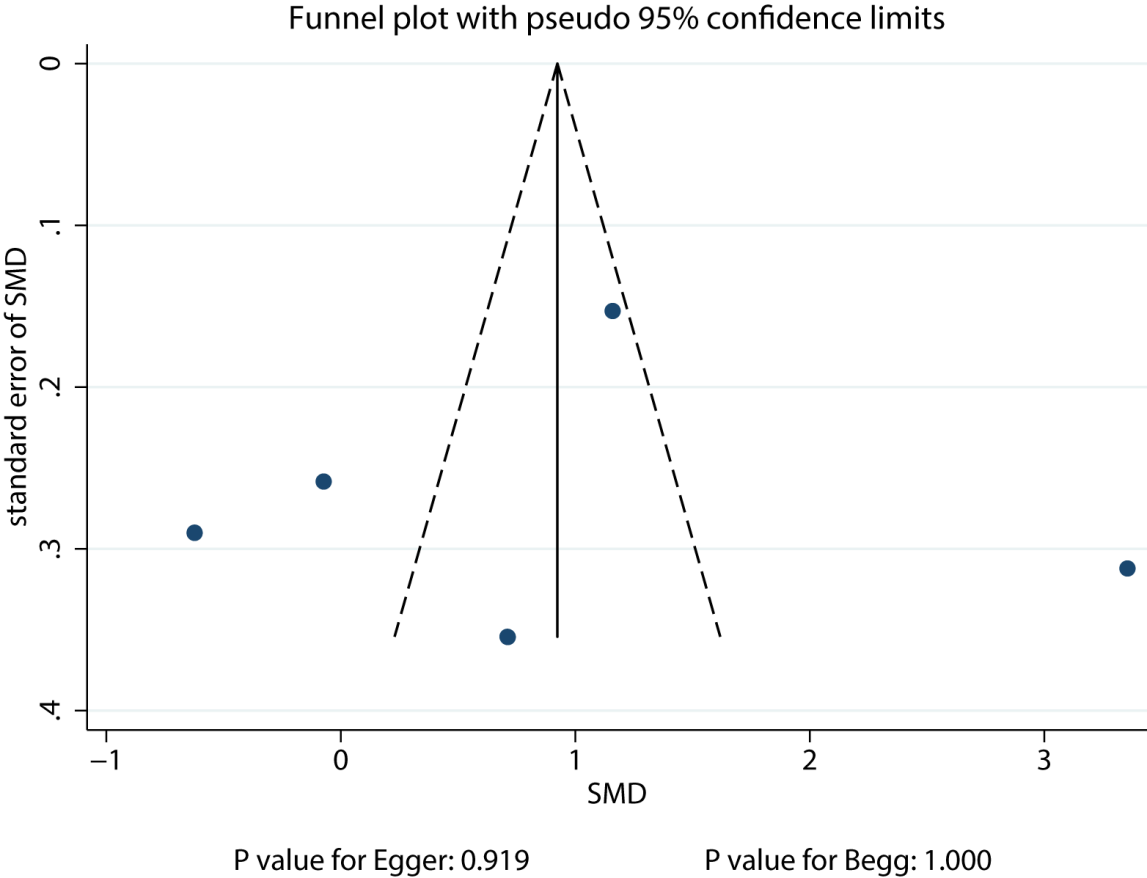


Figure S14. Trim and fill for SOD


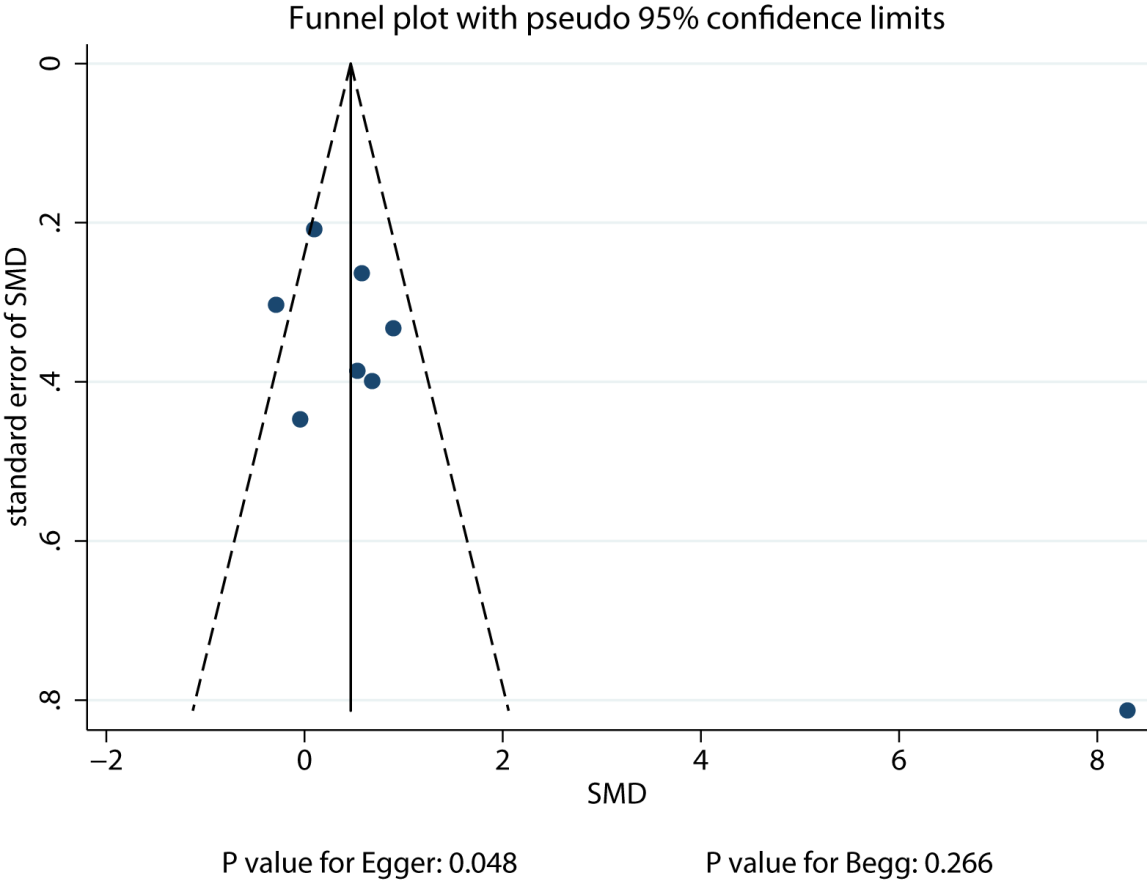


Figure S15. Funnel plot for BMI


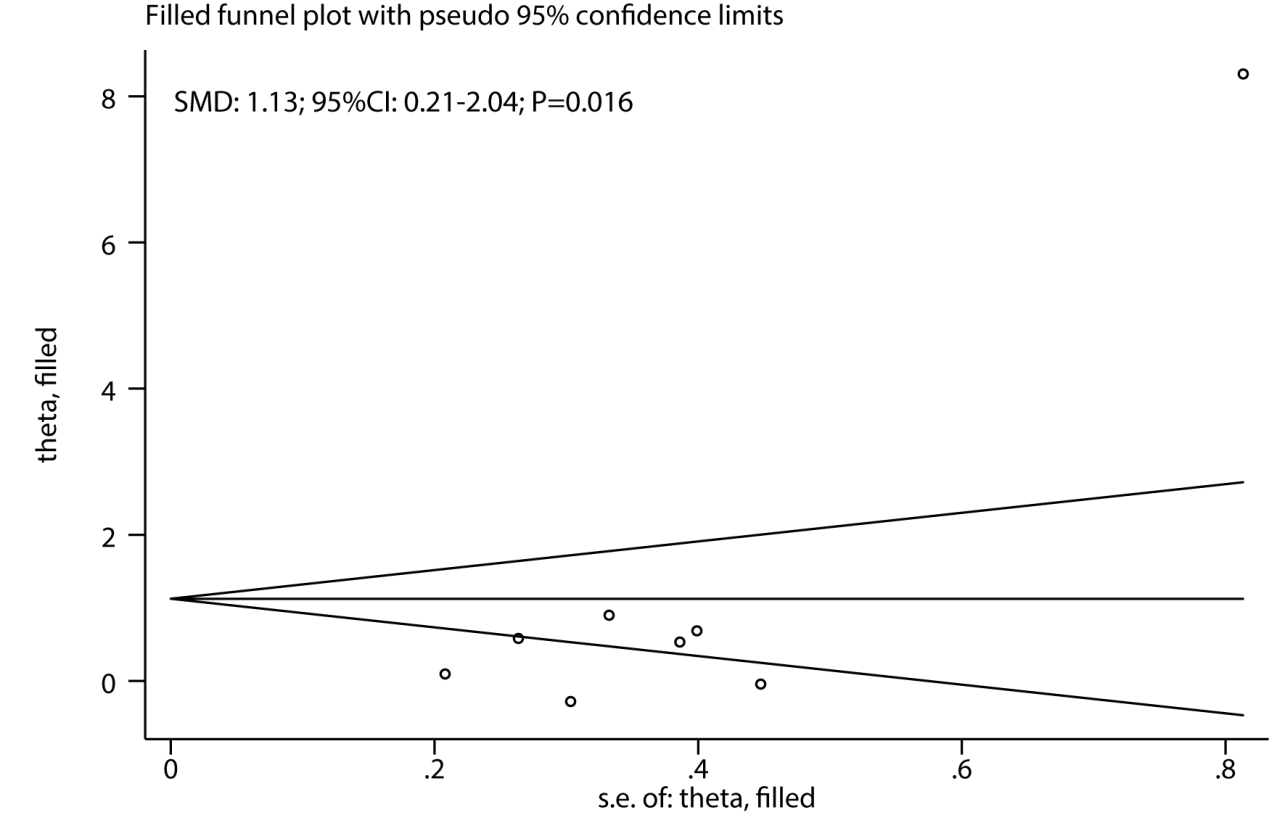


Figure S16. Trim and fill for BMI
